# Acclimation temperature influences phage susceptibility in a toxin-producing strain of *Microcystis aeruginosa*

**DOI:** 10.1128/spectrum.03379-25

**Published:** 2026-06-15

**Authors:** Kennedi M. Hambrick, Laura E. Smith, Robbie M. Martin, Bofan Wei, Raunak Dey, Gregory L. Boyer, Joshua S. Weitz, David Talmy, Steven W. Wilhelm, Erik R. Zinser

**Affiliations:** 1 Department of Microbiology, The University of Tennessee 189504, Knoxville, Tennessee, USA; 2 Department of Chemistry, State University of New York College of Environmental Science and Forestry, Syracuse, New York, USA; 3 Department of Physics, University of Maryland 248530 , College Park, Maryland, USA; 4 Department of Biology, University of Maryland 171291 , College Park, Maryland, USA; 5 University of Maryland Institute for Health Computing 1068 , North Bethesda, Maryland, USA; Universidad Nacional Autonoma de Mexico - Campus Morelos, Cuernavaca, Mexico

**Keywords:** *Microcystis aeruginosa*, cyanophages, temperature, microcystin

## Abstract

*Microcystis aeruginosa* is a freshwater cyanobacterium known for its role in the formation of harmful algal blooms. However, less well characterized are the host-phage interactions that occur in these toxic blooms. Here, we describe the relationship between a microcystin-producing strain of *M. aeruginosa,* NIES-298, and a lytic bacteriophage Ma-LMM01. In this study, we explored how host pre-acclimation to different temperature conditions influenced sensitivity to subsequent infection by Ma-LMM01. We focused on 19°C, a cooler temperature often associated with microcystin-producing blooms in spring/early summer, and 26°C, a warmer temperature often associated with non-microcystin-producing blooms of late summer. *M. aeruginosa* cultures pre-acclimated to 26°C were completely lysed whether infection occurred at 26°C or 19°C. In contrast, 19°C-acclimated *M. aeruginosa* grew and resisted infection regardless of infection temperature. We next formulated a mathematical model to investigate the key parameters driving these temperature dynamics. Our modeling suggested both 19°C- and 26°C-acclimated hosts contain resistant subpopulations of comparable abundance but with different degrees of resistance. Specifically, we found the 19°C-acclimated host subpopulation was substantially more resistant to phage infection than the 26°C-acclimated host subpopulation, which was only partially resistant. Overall, our observations demonstrate that *M. aeruginosa* NIES-298 acclimation to 19°C played a role in the development of resistance to cyanophage Ma-LMM01. These findings suggest new directions to explore the link between physiological changes and infection outcomes for microcystin-producing cyanobacteria in changing environments.

**IMPORTANCE:**

Harmful cyanobacterial blooms frequently develop in aquatic systems, causing significant ecological and commercial impact, motivating research into factors influencing bloom formation, persistence, and toxicity. Prior studies identified several contributors to the “life cycle” of a toxic bloom, including abiotic influences, community structure, and host-viral interactions. This paper explores two of these factors, temperature and viral-host interactions, on the growth and survival of *Microcystis aeruginosa* NIES-298. We report the first observation that the acclimation temperature of *M. aeruginosa* influences phage susceptibility, and through mathematical modeling, explore resistant subpopulation dynamics. We observed that cells acclimated to cold temperatures, such as those experienced in spring/early summer, were resistant to phage, while those acclimated to warmer temperatures, such as those experienced in late summer, were not. This work contributes to the fields of freshwater ecology and microbial physiology by advancing our understanding of the interplay between host-phage interactions and abiotic influences in toxic algal bloom formation.

## INTRODUCTION

The cyanobacterium *Microcystis* is a major contributor to toxic algal blooms in freshwater systems across the globe, such as Lake Erie and Lake Tai (1, 2). Through production of the hepatotoxic secondary metabolite, microcystin, *Microcystis* can contaminate drinking water and lead to negative health effects, including fever, skin rash, and liver damage (3). This qualifies toxic algal blooms as a public health risk and threat to the global freshwater supply (4). Over the past few decades, the occurrence and toxicity of toxic algal blooms have increased, likely due to eutrophication and rising temperatures (5). Abiotic and biotic influences on microbial diversity (6, 7), community interactions (8), and successions in these freshwater systems (9) have all been characterized in recent studies. However, less well characterized are the host-phage interactions in these toxic blooms.

Phages are abundant in the environment, and fresh waters are estimated to contain approximately 1.76 × 10^27^ virus-like particles (10). Within the past two decades, several cyanophages (phages infecting cyanobacteria) (11) have been identified that infect and lyse *M. aeruginosa,* including the Ma-LMM01 phage isolated from Lake Mikata, Japan, and the MaMV-DC phage isolated from Lake Dianchi, China (12, 13). While *Microcystis* phage shows a lytic lifecycle in laboratory settings (12, 14), it has been suggested that lysogeny is a possible infection state as *Microcystis* phage genomes, such as Ma-LMM01, encode genes proposed to be used in lysogeny (15). In the environment, *Microcystis* phage may undergo both lytic and lysogenic states, as previous research has shown changes in *Microcystis* phage lytic and lysogenic gene expression, which may be tied to environmental factors including dissolved solids, pH, and nutrient availability (15). Cyanophages, such as those that infect *Microcystis,* are implicated in the observed composition shifts and eventual collapses of cyanobacterial blooms (16, 17). Despite these crashes, there is never a true extinction of the *M. aeruginosa* population in the environment, as blooms return every year (18).

The observation of concurrent phage and bloom-forming cyanobacteria suggests different potential mechanisms enabling coexistence. Phage defense systems are present in *M. aeruginosa*, and they play underexplored roles in the dynamic ecologies of host and phage. The array of *M. aeruginosa* defenses includes CRISPR/Cas and an exceptionally high number of restriction modification systems (19–21). Indirect evidence suggests that another antiphage mechanism may exist in the form of toxic secondary metabolites, whose production is tied to temperature (22, 23).

*M. aeruginosa* NIES-298 synthesizes a broad spectrum of secondary metabolites, including the hepatotoxic compound originally known as “fast death factor,” but now as microcystin (24). This compound is encoded by the microcystin synthetase gene cluster, which is made up of 10 ORFs (25). There remains a broad series of hypotheses concerning the potential role of microcystin in cellular metabolism (26), with some level of oxidative stress response being the most popular (27, 28). While the role(s) of this secondary metabolite have not been fully characterized, cyanophage activity can be correlated with abundance shifts in microcystin-producing strains, thus allowing one to speculate a role for microcystin as an anti-phage defense mechanism. Specifically, it has been shown that spring/early summer blooms (*T* ~18°C) have increased microcystin concentrations and lower cyanobacterial and viral abundances compared to late summer blooms (*T* ~26°C), which have decreased microcystin concentrations and higher cyanobacterial and viral abundances (22, 23).

In the present study, we manipulated temperature before and during infection to determine which timeframe was critical for phage resistance of the host. This served to assess both (i) temperature as a general contributor and (ii) microcystin concentration on resistance. The phage-host system utilized (Ma-LMM01/NIES-298) lacks genetic tools, but we noted that, for genetically tractable (but lacking a phage isolate) PCC 7806 (29), growth at low temperature resulted in a higher microcystin concentration relative to high temperature; hence, pre-acclimation temperature could be used to vary microcystin per cell prior to infection. Chemostat cultures were first pre-acclimated in the absence of phage to temperatures that established either high (19°C) or low (26°C) concentrations of intracellular toxin. In the context of this manuscript, the term pre-acclimation will define the period of adaptation of the *M. aeruginosa* host to varying temperature conditions in chemostat culture prior to exposure to phage. Culture aliquots subsequently challenged with phage at both high and low temperatures indicated that pre-acclimation (i.e., temperature history and toxin content) was a major driver of resistance, rather than instantaneous temperature.

## MATERIALS AND METHODS

### Chemostat set up and maintenance

Biological duplicate 1 L continuous cultures (chemostats) were inoculated with 8 mL of axenic *M. aeruginosa* NIES-298 in CT medium modified for continuous cultures at 26°C and maintained until steady state (29, 30). The final nitrogen (N) concentration was 323 μM supplied as a mix of Ca(NO_3_)_2_ and KNO_3_ with a N molar ratio of 1.37:1 (29). The final phosphorus (P) concentration was 16.3 μM supplied as K_2_HPO_4_. This resulted in a final N:P ratio of 19.7. Chemostat design and construction followed methods published previously (29). Chemostats were maintained with a dilution rate of 0.25 under 24 h continuous light at 50 μmols photons m^−2^s^−1^ throughout the entirety of the experiment. Both chemostats were housed in the same incubator throughout the entirety of the experiment to minimize differences between replicates. To account for temperature deviations that may have occurred, after day 10, chemostat incubation temperature was measured through the remainder of the experiment with a HOBO TidbiT Temperature Logger (Onset Computer Corporation) set to measure every 15 min (Fig. S1).

After approximately 3 weeks of chemostat incubation, samples were collected every 1–3 days for intracellular microcystin quantification and cell abundance (see below). Once chemostats maintained cell densities ± 7% for three consecutive sample points, as measured by flow cytometry, they were considered at steady state. Once steady state was reached, samples were collected for batch culture infectivity experiments as well as for intracellular microcystin quantification and cell abundance. Incubation temperatures were then lowered to 19°C, and the chemostats were allowed to re-acclimate. Daily measurements of cell concentration restarted 1 week after the change to 19°C to determine the reestablishment of steady state. After acclimation to 19°C for 10 days, samples were again collected to test sensitivity to infection.

### Lysate preparation

The cyanophage Ma-LMM01 was provided by Dr. Jozef Nissimov (University of Waterloo, Canada). A fresh batch of Ma-LMM01 lysate (50 mL) was prepared and used for all infections to control for any variations in the phage population. Briefly, batch culture mid-log phase *M. aeruginosa* NIES-298 was inoculated with a previous stock of Ma-LMM01 lysate and incubated at 26°C under continuous light at 50 μmols photons m^−2^s^−1^. Complete lysis was defined as a total loss of green pigmentation of the culture. The resulting lysate was filter sterilized with a Nalgene 0.2 µm syringe filter with a surfactant-free cellulose acetate membrane (Thermo Scientific). To account for any fertilization effect of lysate addition (i.e., stimulation from lysate materials that were not viral), a portion of the 0.2 µm filter-sterilized lysate was filter sterilized with an Anotop 0.02 µm Syringe Filter (Anotop). Lysates were stored at 4°C until immediately prior to use in infectivity experiments. This 0.02 µm filter-sterilized lysate was challenged against uninfected *M. aeruginosa* NIES-298 cells and compared to uninfected *M. aeruginosa* NIES-298 cells that were maintained with no additional additive (see below). Additionally, it should be noted that as the phage was propagated at 26°C, they may be potentially better able to infect *M. aeruginosa* host that was pre-acclimated to 26°C rather than 19°C.

### Infection experiments

Infectivity assays were performed in batch culture in 50 mL tubes using temperature-acclimated continuous culture as inoculum. To perform infectivity experiments, 40 subsamples of the acclimated chemostat culture were collected and used to test infectivity under low toxin (26°C, *n* = 20) or high toxin (19°C, *n* = 20) conditions. Additionally, 24 tubes (*n* = 12 per acclimation treatment) were maintained as uninfected controls. To establish each replicate used in the infectivity assay, 15 mL of temperature-acclimated continuous culture host was added to 15 mL of standard CT medium (30) for a final concentration of approximately 1.4 × 10^6^ cells mL^−1^. An infectivity assay consisted of 20 replicates (tubes) that were infected with phage and 12 replicates (tubes) serving as uninfected controls. Of these, 10 infected biological replicates and 6 controls were incubated at 26°C, and the others at 19°C. The infectivity assay was simultaneously incubated at two temperatures (19°C and 26°C) to distinguish between the influence of pre-acclimation temperature (which establishes the cellular microcystin concentration) and the temperature at which the infection is allowed to proceed. Tubes assigned to test infectivity were infected with 1 mL Ma-LMM01 for an average phage particle:cyanobacterium ratio of 12. Tubes assigned as uninfected controls had either 1 mL of 0.02 µm filter-sterilized phage lysate added (*n* = 8) or no additional additives (*n* = 16). As there was not a large difference in growth between uninfected controls within temperature treatments, irrespective of 0.02 µm filter-sterilized lysate addition, these two control groups were averaged together (Fig. S2; Fig. 2). Cultures were maintained under 24 h continuous light at 50 μmols photons m^−2^s^−1^ throughout the entirety of the infection experiment.

It has previously been shown that the microcystin quota in *M. aeruginosa* strain PCC7806 has a 48–72 h temporal lag correlation to *mcy* expression (29), demonstrating that acclimated cells maintain toxin concentrations for several days after shifting to a new temperature. Therefore, to distinguish between temperature effects on acclimation—which establishes microcystin concentration—versus effects on infection, each infection assay was completed at both 19°C and 26°C (±3°C) for each acclimated culture (19°C and 26°C). It should be noted that while the goal was to perform an infection assay of both acclimated cultures at 26°C, a fluctuation in incubator temperature led to the 26°C-acclimated host undergoing an infection assay at an average temperature of 23°C. Critically, the phage was intentionally absent during the acclimation stage.

### Microcystin quantification

Prior to infection, pre-acclimated *M. aeruginosa* NIES-298 microcystin concentrations were quantified utilizing liquid chromatography-mass spectroscopy (LC-MS) as outlined in detail at *protocols.io* (31). Steady state *M. aeruginosa* cells (50 mL) were collected from each chemostat (average cell concentration of 2.7 × 10^6^ cells mL^−1^) by vacuum filtration onto 47 mm glass fiber filters (Advantec) and flash frozen in liquid nitrogen. The resultant filters were stored at −80°C until shipment to Syracuse, NY, for processing. There, the filters were extracted in 50% methanol using ultrasound and analyzed by LC-MS. Twenty-two microcystin congeners (RR, dRR, mRR, H4YR, hYR, YR, LR, mLR, zLR, dLR, meLR, AR, FR, WR, LA, dLA, mLA, LL, LY, LW, LF, and WR) were screened, and total microcystin concentrations are reported as the sum of all congeners. Microcystin content for each chemostat on the day of infection experiments was determined by the average of technical duplicates. Microcystin content for each temperature treatment was averaged from individually measured biological replicates.

### Host and viral enumeration

*M. aeruginosa* NIES-298 and Ma-LMM01 abundances were enumerated via flow cytometry on a Beckman Coulter Cytoflex S, equipped with a 50 mW blue laser (488 nm) and a violet laser (405 nm) (32). Measurements were made once daily throughout the length of the infection experiment (8 days). *M. aeruginosa* cell abundances were measured using forward light scatter and Peridinin-Chlorophyll-Protein autofluorescence (PerCP, 690/50) for triplicate 250 µL technical replicates for each sample. Ma-LMM01 virus particles were enumerated by first fixing 600 µL of Ma-LMM01 supernatant from each treatment in 25 µL of 25% glutaraldehyde (final concentration 2.5%) and incubating at room temperature in the dark for 15 min. Fixed samples were then stored at 4°C until processing. Next, the fixed sample was stained with 3 µL 100× SYBR Gold (Invitrogen), incubated at room temperature in the dark for 10 min, and then incubated at 80°C for 10 min. After staining, duplicate 250 µL technical replicates for each sample were enumerated utilizing the violet side scatter and FITC (525/40) channel of the flow cytometer. Fixed and stained CT medium was used as a negative control to determine background interference, and Milli-Q water was used between wells to prevent signal carryover. Abundance for individual samples was determined by averaging the technical replicates.

### Determination of secondary metabolite expression

Biosynthetic gene clusters in the genomes of *Microcystis aeruginosa* PCC7806SL (accession NZ_CP020771.1) and *Microcystis aeruginosa* NIES-298 (accession NZ_CP184724.1) were identified via antiSMASH (v.7.1.0) (33). Detection was set to “strict,” and all other settings were left as default. For gene clusters that overlapped in the two strains (aside from terpenes), cold temperature gene expression data were extracted from *M. aeruginosa* PCC7806 and *M. aeruginosa* PCC7806 Δ*mcyB* normalized transcriptome libraries (34). Methods for transcriptome library collection, preparation, and read mapping can be found elsewhere (34).

### Data analysis

A one-way analysis of variance was performed to determine significance in intracellular microcystin concentration, Ma-LMM01 viral abundance, and host abundance between acclimation treatments. Individual samples were quantified using technical replicates, and averages for each treatment were taken across biological replicates within that treatment.

### Mathematical model

#### Mathematical model description

To explore the effects of temperature on the *M. aeruginosa* host and Ma-LMM01 viral interaction, two models were developed, a lytic population model and a resistant subpopulation model. The lytic population model approximated the host-phage interaction with the underlying assumption that all hosts are susceptible to the phage. In contrast, the resistant subpopulation model assumed the existence of a rare initial subpopulation of resistant hosts within the culture dominated by susceptible hosts.

The lytic and resistant subpopulation models both define the rate of change of a susceptible host abundance as the difference between net host growth and recovery, and infection:


(1) $$\frac{dA_{S}}{dt}=\underset{\text{host growth }}{\underbrace{\mu A_{S}}}+\underset{\text{recovery }}{\underbrace{\lambda_{S}I_{S}\left(1-P_{R,S}\right)}}-\underset{\text{infection }}{\underbrace{\varphi_{S}A_{S}V}}$$

where μ is the algal host growth rate (day^−1^), $A_{S}$ is the susceptible algal host (cells mL^−1^), $\varphi_{S}$ is the viral infection rate of the susceptible algal host (mL virus^−1^ day^−1^), and *V* is the abundance of free phage (virus mL^−1^). Note that all parameters and variables used in this study are listed in Tables 1 and 2. Lysis of an infected susceptible host population *I_S_* (cells mL^−1^) proceeds with lysis rate $\lambda_{S}$ (day^−1^), and the proportion of lysis events that fail leading to recovery is represented by ${(1-P}_{R,S})$. Additionally, host saturation was not explicitly included as the cell density for infected host was less than the uninfected control, with the only exception being the end of the 19°C-acclimated host experiment in which the cell density of the infected host was similar to the uninfected control; however, it was clear that growth was exponential during this time (Fig. 2). The rate of change of an infected host abundance is defined as:


(2) $$\frac{dI_{S}}{dt}=\underset{\text{infection }}{\underbrace{\varphi_{S}A_{S}V}}-\underset{\text{lysis }}{\underbrace{\lambda_{S}I_{S}}}$$

**TABLE 1:** State variables utilized in the mathematical models^*a*^

Symbol	Unit	Definition
*A_S_*	cells mL^−1^	Susceptible algal host
*I_S_*	cells mL^−1^	Infected susceptible algal host
*V*	virus mL^−1^	Free virus
*A_R_*	cells mL^−1^	Resistant algal host
*I_R_*	cells mL^−1^	Infected resistant algal host

^*a*^ The state variables: *A_S_* (susceptible algal host), *I_S_* (infected susceptible algal host), and *V *(free virus) were utilized in both population models. The state variables *A_R_* (resistant algal host) and *I_R _*(infected resistant algal host) were only used within the resistant subpopulation models.

**TABLE 2:** Parameters used in mathematical models^*a*^

Symbol	Unit	Definition	Description
*A_S,0_*	cells mL^−1^	Susceptible host initial abundance	In the LPM, *A_S,0_* was experimentally derived and fixed. In the RSM, *A_S,0_* was determined by subtracting *A_R,0_* from the experimentally derived algal host abundance.
*I_S,0_*	cells mL^−1^	Infected susceptible host initial abundance	In the LPM and the RSM, *I_S,0_* was fixed at 0 cells mL^−1^ as it was assumed no cells were actively infected at *t*_0_.
*V_0_*	virus mL^−1^	Free virus initial abundance	In the LPM and the RSM*, V_0_* was fixed and experimentally derived.
*A_R,0_*	cells mL^−1^	Resistant host initial abundance	In the LPM, parameter absent. In the RSM, *A_R,0_* = 50,000 cells mL^−1^ (26°C-acclimated host infected at 26°C); 100,000 cells mL^−1^ (26°C-acclimated host infected at 19°C); and *A_R,0_ =* 220,000 cells mL^−1^ (19°C-acclimated hosts).
*I_R,0_*	cells mL^−1^	Infected resistant host initial abundance	In the LPM, parameter absent. In the RSM, *I_R,0_* was fixed at 0 cells mL^−1^ as it was assumed no cells were actively infected at *t*_0_.
*μ*	d^−1^	Host growth rate	In the LPM, *μ* = 0.36 d^−1^ (26°C-acclimated hosts) and *μ* = 0.24 d^−1^ (19°C-acclimated hosts). In the RSM, *μ* = 0.36 d^−1^ (26°C-acclimated hosts) and *μ* = 0.44 d^−1^ (19°C-acclimated hosts).
*φ_S_*	mL virus^−1^ d^−1^	Viral infection rate of susceptible host	In the LPM, *φ_S_* = 6 × 10^−8^ mL virus^−1^ d^−1^ (26°C-acclimated hosts) and *φ_S_* = 6 × 10^−9^ mL virus^−1^ d^−1^ (19°C-acclimated hosts). In the RSM, *φ_S_* = 6 × 10^−8^ mL virus^−1^ d^−1^.
φ*_R_*	mL virus^−1^ d^−1^	Viral infection rate of resistant host	In the LPM, parameter absent. In the RSM, *φ_R_* = 3 × 10^−9^ mL virus^−1^ d^−1^.
λ*_S_*	d^−1^	Lysis rate of susceptible host	In the LPM and the RSM, *λ_S_* = 0.65 d^−1^.
*λ_R_*	d^−1^	Lysis rate of resistant host	In the LPM, parameter absent. In the RSM, *λ_R_* = 1 d^−1^ (26°C-acclimated hosts) and *λ_R_* = 4 d^−1^ (19°C-acclimated host infected at 26°C); 3.5 d^−1^ (19°C-acclimated host infected at 19°C).
*β_S_*	virus cell^−1^	Phage burst size from susceptible algal host	In the LPM and RSM, β*_S_* = 90 virus cell^−1^.
*β_R_*	virus cell^−1^	Phage burst size from resistant algal host	In the LPM, parameter absent. In the RSM, β*_R_* = 60 virus cell^−1^ (26°C-acclimated hosts) and β*_R_* = 150 virus cell^−1^ (19°C-acclimated host infected at 26°C); 180 virus cell^−1^ (19°C-acclimated host infected at 19°C).
*P_R,S_*		Proportion of susceptible host lysis events that succeed	In the LPM, *P_R,S_ =* 0.5 (26°C-acclimated host infected at 26°C); 0.4 (26°C-acclimated host infected at 19°C); and *P_R,S_ =* 1 (19°C-acclimated host infected at 26°C); 0.9 (19°C-acclimated host infected at 19°C). In the RSM, *P_R,S_ =* 0.60.
*P_R,R_*		Proportion of resistant host lysis events that succeed	In the LPM, parameter absent. In the RSM, *P_R,R_ =* 0.4 (26°C-acclimated host infected at 26°C); 0.6 (26°C-acclimated host infected at 19°C); and 0.2 (19°C-acclimated hosts).

^*a*^ This table lists the parameters used within the models described in this text. The parameters *A_S,0_*, *I_S,0_*, *V_0_*, *μ*, *φ_S_*, *λ_S_*, *β_S_*, and *P_R,S_* were used within both lytic population model (LPM) and resistant subpopulation model (RSM). The parameters *A_R,0_*, *I_R,0_*, *φ_R_*, *λ_R_*, *β_R_*, and *P_R,R_* were used only within the RSM. Parameter values listed represent those used for a given temperature-acclimated host treatment regardless of infection temperature unless stated. Additionally, parameter values were equal for both 26°C- and 19°C-acclimated hosts unless otherwise stated.

where *λ_S_* is the infected susceptible host lysis rate (day^−1^), and *I_S_* is the infected susceptible algal host (cells mL^−1^). A natural death term was not explicitly included for the infected host population as it was assumed to be far less than that imposed by *λ_S_*. Lastly, the rate of change of an abundance of free virus can be described as:


(3) $$\frac{dV}{dt}=\underset{\text{lysis }}{\underbrace{\beta_{S}\lambda_{S}I_{S}P_{R,S}}}-\underset{\text{infection }}{\underbrace{\varphi_{S}A_{S}V}}$$

where *β_S_* is the burst size of phage from the infected susceptible host (virus cell^−1^). We assume negligible viral decay in the experiment described within as shown by an increase in net viral abundance for both host acclimation treatments in Fig. 2; therefore, we exclude this term from our model. However, as viral decay is observed in natural systems, we assert this model is best used for experiments in which viral decay is not observed.

Additionally, a resistant subpopulation model was developed by adding a resistant algal subpopulation to the lytic model. The rate of change of a resistant host abundance can be defined as the difference between net host growth and recovery, and infection:


(4) $$\frac{dA_{R}}{dt}=\underset{\text{host growth }}{\underbrace{\mu A_{R}}}+\underset{\text{recovery }}{\underbrace{\lambda_{R}I_{R}\left(1-P_{R,R}\right)}}-\underset{\text{infection }}{\underbrace{\varphi_{R}A_{R}V}}$$

where $A_{R}$ is the resistant algal host (cells mL^−1^), and $\varphi_{R}$ is the viral infection rate of the resistant algal host (mL virus^−1^ day^−1^). Lysis of an infected resistant algal host (cells mL^−1^) proceeds with rate $\lambda_{R}$ (day^−1^), and the proportion of lysis events leading to host recovery is $\left(1-P_{R,R}\right)$. The rate of change of an abundance of infected host cells derived from the resistant host population is defined as:


(5) $$\frac{dI_{R}}{dt}=\underset{\text{infection }}{\underbrace{\varphi_{R}A_{R}V}}-\underset{\text{lysis }}{\underbrace{\lambda_{R}I_{R}}}$$

where $\lambda_{R}$ is the infected resistant host lysis rate (day^−1^), and *I_R_* is the infected resistant algal host (cells mL^−1^). Finally, the rate of change of an abundance of free virus can be described as:


(6) $$\frac{dV}{dt}=\underset{\text{lysis }}{\underbrace{\beta_{S}\lambda_{S}I_{S}P_{R,S}+\beta_{R}\lambda_{R}I_{R}P_{R,R}}}-\underset{\text{infection }}{\underbrace{(\varphi_{S}A_{S}+\varphi_{R}A_{R})V}}$$

where $\beta_{R}$ is the phage burst size from the infected resistant host (virus cell^−1^). As mentioned previously, we will assume negligible viral decay and thus exclude this term from our model equation 6.

All mathematical modeling was conducted in Spyder using Python 3.9.18. All code along with corresponding data can be accessed at https://github.com/kennedihambrick/NIES298infection.git.

#### Model-data comparison

Model-data comparisons were quantified with *R*^2^ (coefficient of determination) and a likelihood using the log-transformed model and data. The likelihood (L) was calculated with:


(7) $$L=exp\left(-\frac{1}{\sigma }\sum_{i=1,n}{\left(y_{i,d}-y_{i,m}\right)}^{2}\right)$$

where n is the total number of observed host and virus abundances (mL^−1^), $y_{i,d}$ is the log-transformed *i*th data points with corresponding log-transformed $y_{i,d}$ model prediction, and σ is a measure of uncertainty calculated with analysis of triplicate host and duplicate virus abundance data (35). The likelihood function is bounded between 0 and 1. The likelihood and *R*^2^ values had negligible differences regarding best-fit parameters; therefore, only the likelihood values are reported.

#### Parameter quantification and sensitivity analyses

Parameters directly constrained with experimental data are the initial susceptible host ($A_{S,0}$) and virus (*V_0_*) abundance for the lytic population model. The remaining parameters were all determined with a combination of sensitivity analysis and literature data. First, numerous combinations of parameter values for the lytic population model were tested, and the *R*^2^ and likelihood for each of these combinations were calculated. Through this analysis, a base set of parameters was determined. Each parameter was then varied one at a time, and a likelihood was calculated to determine parameter sensitivity (Fig. S3 to S7). For the LPM, the model was highly sensitive to the infection rate ($\varphi_{S}$), host growth rate (*μ*), lysis rate ($\lambda_{S}$), and the proportion of successful lysis events (*P_R,S_*; Fig. S3, S4, S6 and S7) and less sensitive to burst size ($\beta_{S})$; Fig. S5). For the LPM, values of host growth rate and burst sizes (12) that were associated with high likelihood were consistent with literature values for *M. aeruginosa* NIES-298 and Ma-LMM01 (Table 2). Values for infection rate, lysis rate, and the proportion of successful lysis events were selected that were associated with the highest likelihood.

Parameter values for the lytic population model were also used for the resistant subpopulation model, with the one exception that the initial susceptible host abundance ($A_{S,0}$) was adjusted by subtracting the initial condition for the number of resistant hosts ($A_{R,0}$) from the total measured initial host abundance (Table 2). A one-at-a-time parameter sensitivity was also performed for the resistant subpopulation model parameters $\varphi_{R},\beta_{R},\lambda_{R},P_{R,R}$ (Fig. S8 to S12), selecting the value with the highest likelihood in each case. A more thorough sensitivity analysis was also performed by calculating likelihoods for many combinations of the initial concentration of resistant hosts ($A_{R,0})$) and the proportion of resistant host lysis events that succeed ($P_{R,R}$), to explore possible interacting or redundant effects of these parameters on modeled dynamics.

## RESULTS

### *M. aeruginosa* physiology under varying acclimation temperatures

To assess *M. aeruginosa* NIES-298 physiology at varying temperatures, *M. aeruginosa* NIES-298 was acclimated in biological duplicate chemostats sequentially to 26°C then 19°C. We note that to maintain ecological relevance, acclimation temperatures of 26°C and 19°C were chosen to simulate those found during early and late summer blooms in Lake Erie (27, 34). We hypothesized that under chemostat acclimation to 19°C, *M. aeruginosa* NIES-298 would show a decreased steady-state cell abundance, yet increased intracellular microcystin concentration compared to the same strain acclimated to 26°C. These differences have been shown previously for other *M. aeruginosa* strains in both chemostat and batch cultures (27, 34, 36).

**Fig 1:**
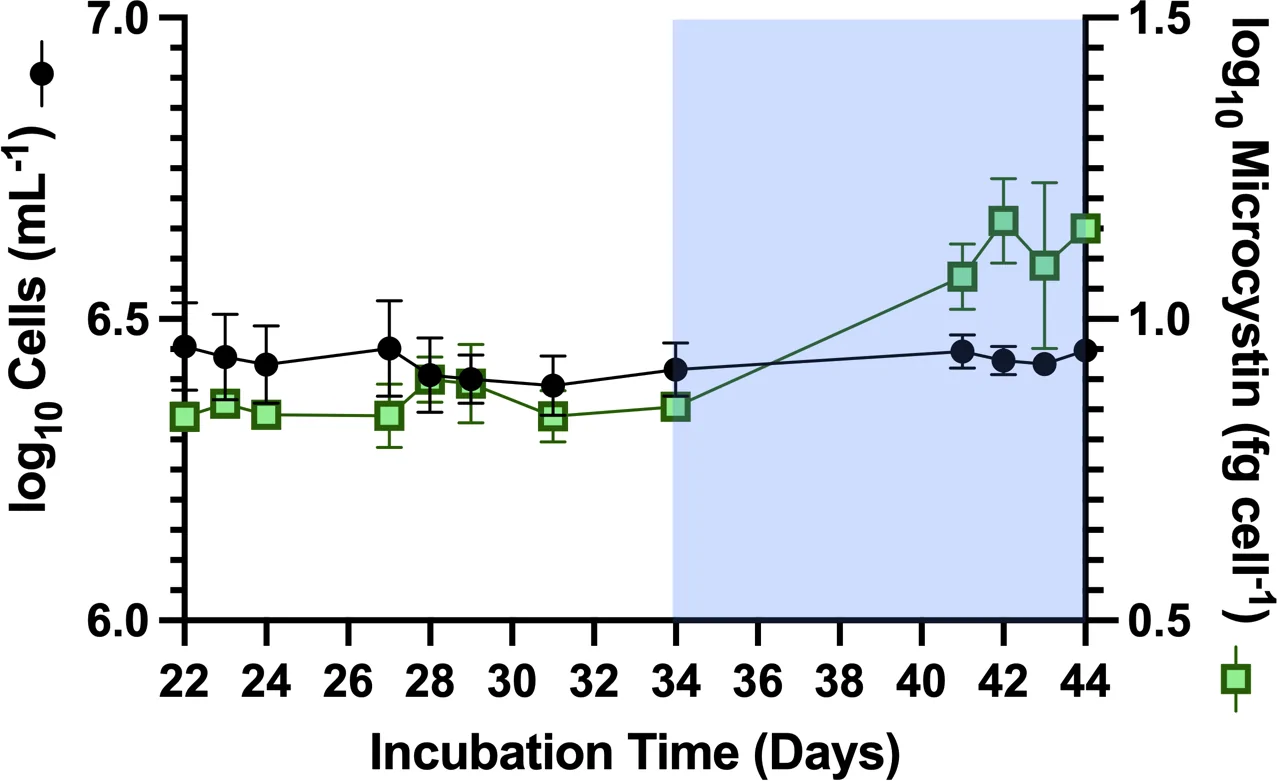
Chemostat *Microcystis aeruginosa* NIES-298 baseline cell concentration and intracellular microcystin concentration. Left y-axis represents chemostat-acclimated *M. aeruginosa* NIES-298 log-transformed cell concentration. Right y-axis represents chemostat-acclimated *M. aeruginosa* NIES-298 log-transformed intracellular microcystin concentration (fg cell^−1^). The x-axis represents chemostat incubation time in days, with measurements starting at chemostat day 22. Blue shading represents days during which chemostat temperature was lowered to 19°C. Black solid circles correspond to *M. aeruginosa* NIES-298 algal cell concentration. Green solid squares correspond to algal intracellular microcystin concentration. Data points indicate mean ± SD.

Steady state conditions were defined as periods where cell concentration did not vary (±7%) on consecutive days: days 22–24 for 26°C and days 41–44 for 19°C (Fig. 1; Table S1). Contrary to expectations, cell concentrations in steady state did not vary between temperature conditions (Fig. 1; Table S1). This indicates that the temperature shift from 26°C to 19°C in chemostat did not substantially alter host growth rate, which opposes results found in previous studies (27, 34, 36). However, the intracellular microcystin concentration was significantly higher (*P* value = 0.043; Table S2) for cells maintained at 19°C versus 26°C (14.13 ± 0.20 versus 7.15 ± 0.02 fg cell^−1^, respectively), consistent with observations in other strains of *M. aeruginosa* (29).

### The effect of temperature acclimation on Ma-LMM01 sensitivity

Temperature can influence infection by both setting host physiology prior to phage exposure and impacting phage-host interactions during the infection process. To assess the relative importance of each temperature effect, we challenged *M. aeruginosa* NIES-298 hosts pre-acclimated to 26°C and 19°C with subsequent phage Ma-LMM01 exposures at both temperatures. The acclimation step lacked exposure to phage, and given that changes in microcystin concentration lag several days after a temperature shift, the acclimation temperature also set the microcystin concentration for the first several days of the subsequent infection stage.

**Fig 2:**
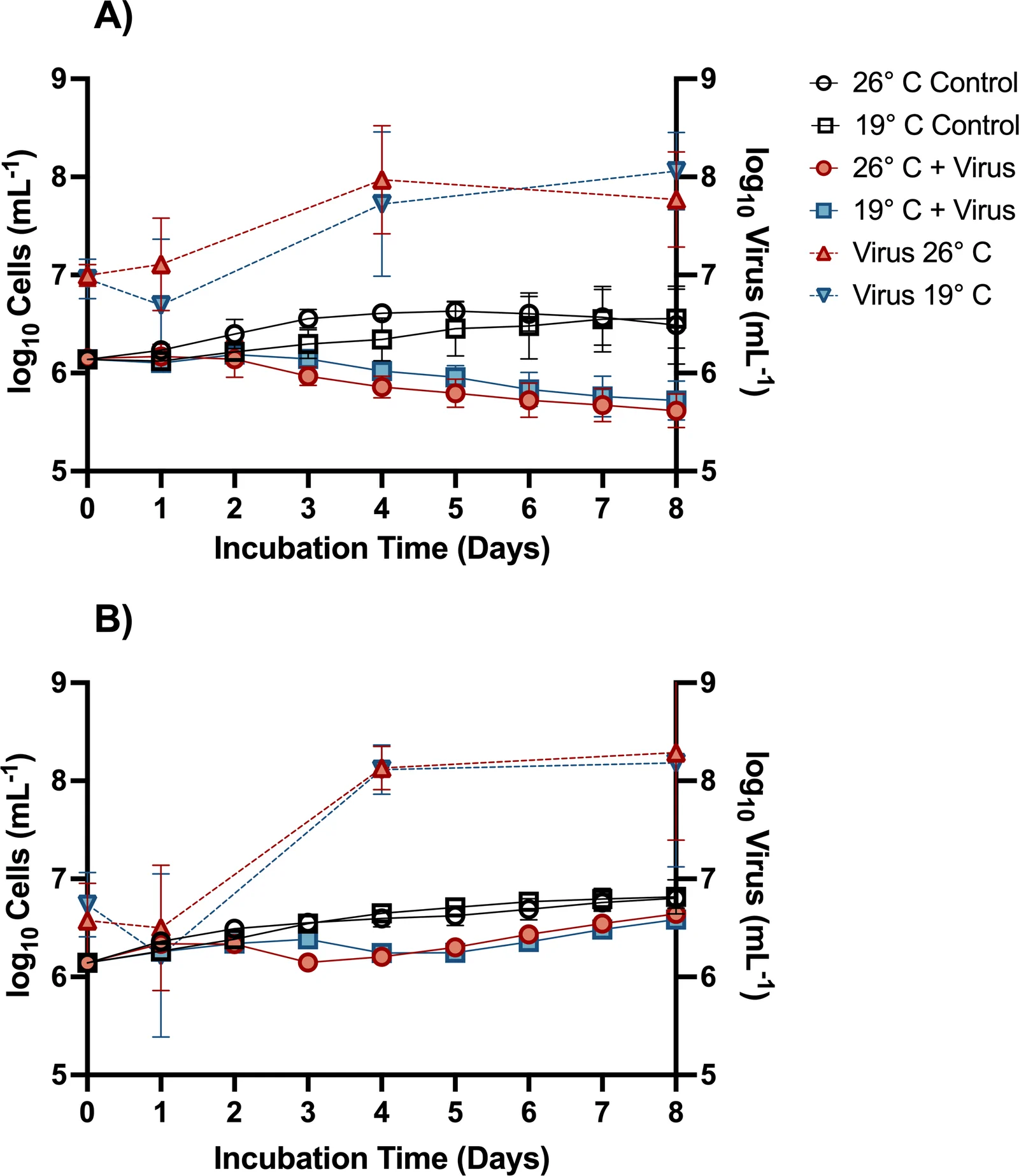
Infection experiment of *Microcystis aeruginosa* NIES-298 infected with cyanophage at time 0. Log-transformed average cell concentrations post-infection of algal host acclimated in chemostat at (**A**) 26°C and (**B**) 19°C. The *x*-axis is incubation time in units of days. The left *y*-axis represents log-transformed *M. aeruginosa* NIES-298 cell concentration. The right *y*-axis represents log-transformed Ma-LMM01 viral abundances. *M. aeruginosa* acclimated at 26°C showed susceptibility to the cyanophage, while *M. aeruginosa* acclimated at 19°C showed resistance to the cyanophage. Host without cyanophage infection at 26°C is represented by the black open circles, host without cyanophage infection at 19°C is represented by the black open squares, host infected with cyanophage at 26°C is represented by red closed circles, and host infected with cyanophage at 19°C is represented by blue closed squares. Additionally, virus particles during infection at 26°C are represented by red triangles with a dotted line, while virus particles during infection at 19°C are represented by blue upside-down triangles with a dotted line. Error bars indicate mean ± SD.

*M. aeruginosa* pre-acclimated in chemostats to 26°C was highly susceptible to lytic infection by the cyanophage regardless of the temperature during phage exposure (Fig. 2A). This was marked by a statistically significant decrease in host abundance 8 days post cyanophage introduction at 26°C (± 3°C; *T*_8_ = 4.45 [±0.52] × 10^5^ cells mL^−1^) compared to the non-infected host (*T*_8_ = 4.01 [±1.02] × 10^6^ cells mL^−1^; Table S3). This trend was also shown in Fig. 2A, as there was a statistically significant decrease in host abundance 8 days post-infection for 26°C-acclimated host infected with cyanophage at 19°C (*T*_8_ = 5.73 [±0.80] × 10^5^ cells mL^−1^) compared with its non-infected control (*T*_8_ = 4.28 [±0.98] × 10^6^ cells mL^−1^). This indicates that the temperature at the time of infection does not alter the outcome of infection of this cyanophage when the host is acclimated at 26°C.

*M. aeruginosa* pre-acclimated in chemostat to 19°C then exposed to cyanophage, however, showed resistance to Ma-LMM01 (Fig. 2B). There was no statistically significant difference in host abundance 8 days post-infection with Ma-LMM01 when infected at both 26°C (*T*_8_ = 4.40 [±0.08] × 10^6^ cells mL^−1^) and 19°C (*T*_8_ = 3.86 [±0.13] × 10^6^ cells mL^−1^) compared to the non-infected host at both 26°C (*T*_8_ = 6.48 [±0.32] × 10^6^ cells mL^−1^) and 19°C (*T*_8_ = 6.95 [±0.46] × 10^6^ cells mL^−1^; Table S4; Fig. 2B). Thus, resistance to the cyanophage was not dependent on temperature during infection (26°C or 19°C; Fig. 2B), but the pre**-**acclimation temperature of the *M. aeruginosa* NIES-298 host played a significant role in the host-phage dynamics of this system.

To further assess the effects of acclimation temperature on virus-host interactions, we quantified the production of Ma-LMM01 particles. Starting on day 4 post-infection, we observed an increase in viral abundance from all infection treatments irrespective of host acclimation temperature or infection temperature (Fig. 2). This increase in viral abundance after day 4 of infection can likely be attributed to repeated rounds of successful infection by Ma-LMM01 and thus the production of progeny phage. Additionally, there was not a significant difference in the number of Ma-LMM01 virus particles produced 8 days post-infection from the 26°C chemostat-acclimated *M. aeruginosa* compared to the 19°C chemostat-acclimated *M. aeruginosa.* This provides strong evidence for a susceptible host subpopulation in the 19°C acclimation treatment alongside a resistant host subpopulation, a possibility we explore with mathematical models.

### Viral lysis drives host-phage dynamics in resistant subpopulation model

**Fig 3:**
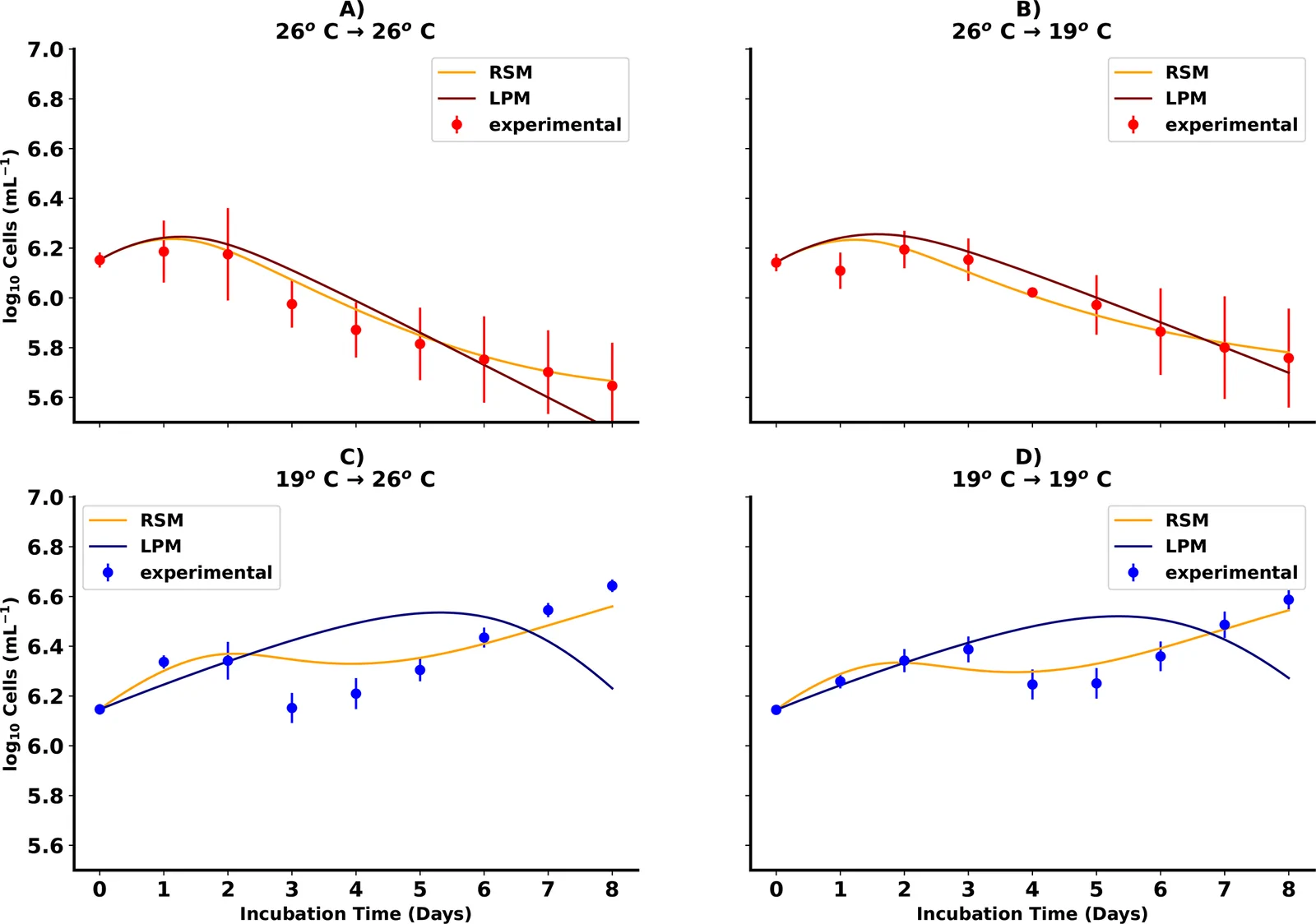
Best model comparisons for the lytic population model and resistant subpopulation model compared to the experimental data. Best fits for the resistant subpopulation model (RSM) and lytic population model (LPM) compared to the experimental data for infection of the (**A**) 26°C-acclimated *M. aeruginosa* host infected at 26°C, (**B**) 26°C-acclimated host infected at 19°C, (**C**) 19°C-acclimated host infected at 26°C, and (**D**) 19°C-acclimated host infected at 19°C. The *x*-axis is time in days. The *y*-axis is log-transformed algal cell abundance (cells mL^−1^). The experimental data are represented by the closed circles. The resistant subpopulation model, which has the highest maximal likelihood, is represented by the gold line. The lytic population model is represented by either a red line (**A and B**) or a blue line (**C and D**). Error bars indicate experimental data mean ± SD.

Infection involves multiple parameters such as host growth rate, infection rate, lysis rate, burst size, etc., all of which could contribute to the different dynamics of infection seen in the empirical data of Fig. 2. To provide insight into which parameter(s) control the *M. aeruginosa* and Ma-LMM01 interaction, we developed and analyzed several models. We first asked if the population dynamics in this host/phage system could be explained with a simple model that assumes a single host and a single viral phenotype, with a lytic infection dynamic. Both 26°C and 19°C lytic models led to predictions of host abundance that increased to a maximum and then declined exponentially, critically lacking the characteristic inflection points evident in the experimental data (Fig. 3). This qualitatively different prediction of host dynamics led us to conclude that the simple lytic population model could not sufficiently capture the dynamics of the experimental data.

The resistant subpopulation model predicted qualitatively different dynamics that were more consistent with the experimental data (Fig. 3). Specifically, for the 26°C acclimation treatment (Fig. 3A and B), the resistant subpopulation model predicted a far more gradual decline in host abundance than the lytic population model, indicating that some form of resistance is present in the warmer condition. Differences between the RSM and the LPM were more pronounced in the 19°C acclimation treatment (Fig. 3C and D), with the RSM recapitulating the early host biomass peak followed by increases in host density. Therefore, it was concluded that a resistant host subpopulation was necessary to model all treatments of the experimental data and that the resistant subpopulation model better characterized this host-phage system.

**Fig 4:**
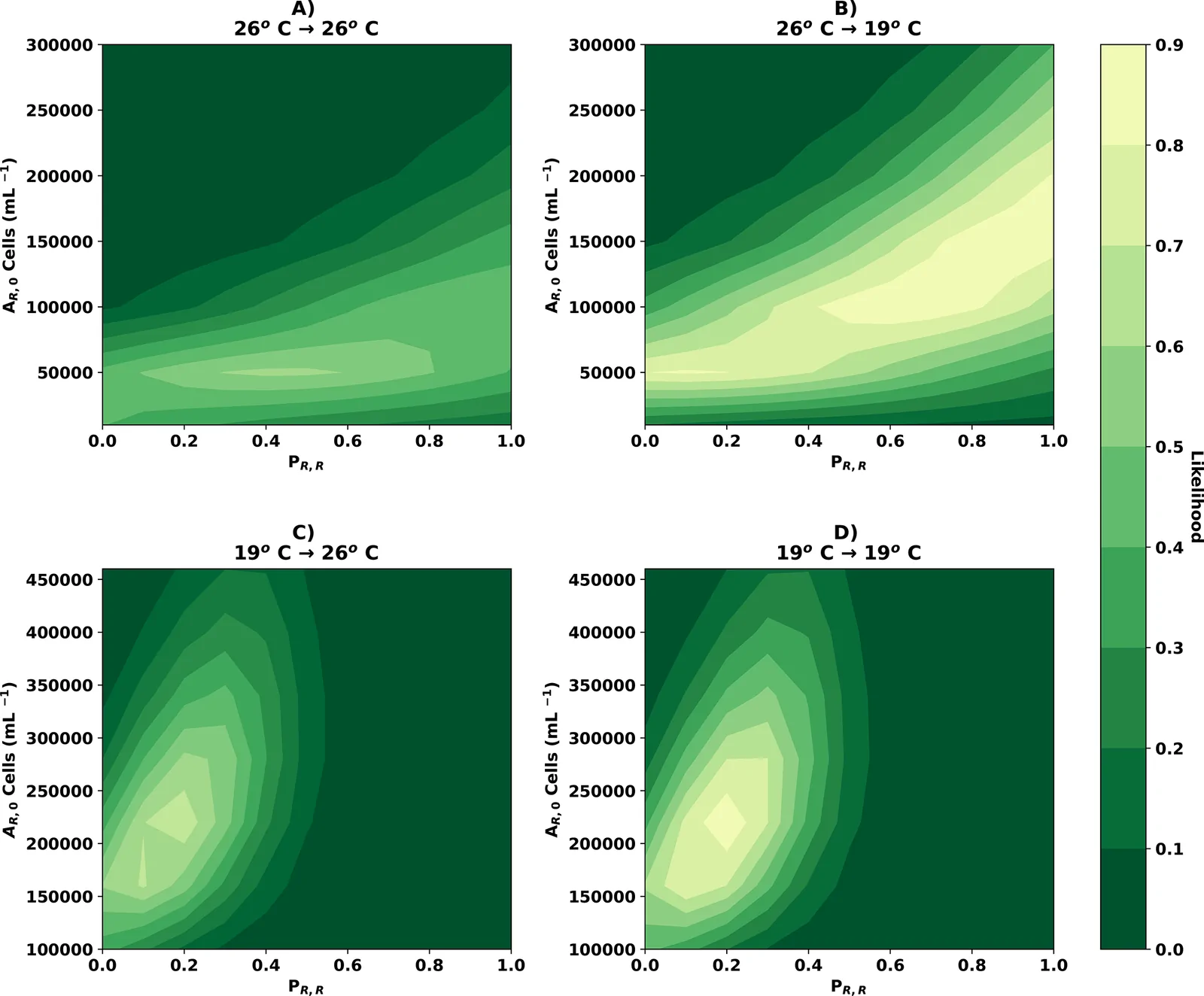
Analysis of model sensitivity to key parameters “*P_R,R_*” (proportion of resistant host lysis events that succeed) and “*A_R,0_*” (initial resistant algal cell concentration). Variance in model fit from the experimental data for (**A**) 26°C-acclimated algal hosts infected with cyanophage at 26°C, (**B**) 26°C-acclimated algal hosts infected with cyanophage at 19°C, (**C**) 19°C-acclimated algal hosts infected with cyanophage at 19°C, and (**D**) 19°C-acclimated algal hosts infected with cyanophage at 19°C. The *x*-axis is the proportion of resistant host lysis events that succeed “*P_R,R_*.” The *y*-axis is the initial resistant algal cell concentration in (cells mL^−1^) and is represented by the initial condition “*A_R,0_*.” The colored scale bar to the right of the subplots represents the corresponding likelihood values, which are a means to measure the uncertainty between the model and the experimental data through a probability distribution.

We next determined if the size of the initial resistant subpopulation, a phenotype of the initial resistant host subpopulation, or an interactive effect between the size and the phenotype was leading to the resistance trend in both our experimental data for the 19°C acclimation treatment and our model. For this, we performed an extensive search of the resistant subpopulation initial condition ($A_{R,0}$) and the proportion of resistant host lysis events that succeed (*P_R,R_*; Fig. 4). The analysis suggested a lower bound *P_R,R_* of ~0.4 for the 26°C (Fig. 4A and B) contrasted with an upper bound on *P_R,R_* of ~0.2 for the 19°C acclimation temperature (Fig. 4C and D). Additionally, it was determined that while the abundance of the initial resistant host population was crucial to improve the model, there was no appreciable change in that parameter between the 26°C and 19°C treatments (Fig. 4), as likelihood was highest at ~0.5–2.2 × 10^5^ cells ml^−1^ (~4%–16% of initial host cells) for both temperatures. Thus, indicating that the abundance of the initial resistant host population was not the main factor driving temperature sensitivity of the host-phage dynamic, but instead it was the inability of the phage to lyse that resistant host.

## DISCUSSION

This study used a combination of experimental observations and mathematical modeling to examine the effect of temperature acclimation on the infection dynamics of the cyanobacterium *Microcystis aeruginosa* NIES-298 by its cyanophage Ma-LMM01. We discuss these results in the context of how a bloom-forming organism that seasonally begins proliferation at or near our cooler temperature (i.e., <19°C) and reaches seasonal biomass maxima at our higher experimental temperature (i.e., ~26°C) (18). We found that *M. aeruginosa* NIES-298 cultures acclimated to 19°C showed greater resistance to phage Ma-LMM01 infection, while cultures pre-acclimated to 26°C showed greater susceptibility. We further demonstrated that the resistance obtained in the 19°C pre-acclimated host was not affected by temperature during infection.

### Evidence for subpopulations

Despite the growth of the 19°C pre-acclimated host in the presence of phage, indicating resistance, the phage was able to reproduce in this system. This phage growth did not impact the growth of the total host population, as it was indistinguishable from growth in the absence of phage. These results indicated that there are at least two subpopulations present in the 19°C pre-acclimation treatments, a susceptible population that is lysed by the phage and a resistant population.

To improve our understanding of how this process played out, we developed a mathematical model with a resistant subpopulation that recapitulated the experimental data from our treatments. This indicated that two subpopulations were present within the community at the time of virus exposure. The 26°C-acclimated treatments showed populations capable of being successfully infected and lysed by the cyanophage (Fig. 3A and B, 4A and B), while the 19°C-acclimated treatments showed both a susceptible population and a resistant subpopulation (Fig. 3C and D, 4C and D). This phenomenon could explain the drop in phage populations in cooler temperature months as *M. aeruginosa* populations shift to be dominated by resistant phenotypes present.

### Mechanism(s) of phage resistance

The mechanism for phage resistance acquired by growth at 19°C, in the absence of phage, is not fully known. However, several hypotheses may explain these observations. One hypothesis is that the greater accumulation of the toxic secondary metabolite microcystin at 19°C confers resistance to phage. Previous studies of other strains indicate that *M. aeruginosa* increases cellular toxin quotas when cells are exposed to a temperature drop from 26°C to 19°C, and the toxin remains within cells for at least 72 h (27, 29, 34). In this study, we found a similar increase in microcystin quota during 19°C versus 26°C acclimation for strain NIES-298. Thus, by exploiting temperature to regulate microcystin in a phage-susceptible host, we observed a direct correlation between microcystin concentration and phage resistance.

How microcystin might protect cells from phage is not understood, but one intriguing possibility involves resource restriction during infection. Upon phage adsorption to a host cell, the virus uses the host’s nutrients and machinery to develop the infection and build progeny phage. These activities often require the repurposing of host cell components to build viral components. However, if the infected virocell cannot obtain nutrients to build progeny phage, the infection is often unsuccessful (37, 38).

A key resource that microcystin may protect from phage is the phycobilisome. The cyanophage Ma-LMM01 encodes for a homolog of the host’s non-bleaching A (NblA) protein, and it has been proposed that the phage NblA enhances degradation of the host phycobilisome, leading to increased production of amino acid precursors and a larger intracellular nitrogen pool for viral protein synthesis (39–41). Notably, microcystin binds to the phycobilisome proteins CpcB and ApcA under high light-induced oxidative stress (42). These observations offer the possibility that microcystin could act as an anti-viral defense in *M. aeruginosa* by binding to the phycobilisome to prevent degradation for nutrient acquisition by Ma-LMM01. While *M. aeruginosa* strains exist that lack the microcystin biosynthetic cluster, these strains are not susceptible to known phages. Therefore, to our knowledge, the only way to vary microcystin concentration in phage-susceptible strains is by manipulating abiotic factors such as temperature. Additionally, future studies look to incorporate nutrient and microcystin dynamics into mathematical models to examine the influence of these parameters on phage dynamics.

We note that in addition to microcystin, there are other secondary metabolites within *Microcystis* whose abundance may also change with temperature. An antiSMASH analysis was performed on strain PCC7806 to provide insight by comparison into which secondary metabolites might be expressed in *M. aeruginosa* NIES-298. *M. aeruginosa* NIES-298 had seven gene clusters in common with PCC7806 (Table S5). Analysis of data from an unrelated experiment with PCC7806 demonstrated that several of these shared gene clusters (i.e., aeruginosin, microcyclamide, and micropeptin) are differentially expressed depending on temperature (Fig. S13). Therefore, it can be cautiously hypothesized that these shared gene clusters are also differentially expressed in *M. aeruginosa* NIES-298. Whether these secondary metabolites provide additional contributions to phage resistance is yet to be determined.

Another, non-mutually exclusive mechanism of temperature-dependent phage resistance may be lysogeny. Lysogens are known to be resistant to superinfection in many microbial genera (43, 44), and so in the present case, lysogeny may help explain our host cell persistence. In this scenario, the cyanophage Ma-LMM01 would infect its host yet form a lysogen as opposed to immediately undertaking a lytic infection. During the lysogenic stage, Ma-LMM01 would remain as an integrated prophage until a signal is received that switches the prophage into the lytic cycle. Whether Ma-LMM01 forms lysogens and if temperature influences the lysogenic-lytic transitions remains to be determined. However, when lysogeny has been invoked in *Microcystis* bloom dynamics, it has come late in the summer season (when temperatures are higher) (15).

Another possibility worth considering is temperature-dependent phage defense expression. *Microcystis* genomes encode dozens of putative defenses, including restriction endonucleases and CRISPR-Cas systems (19–21), whose regulation is not yet known. If, for instance, these defenses are synthesized preferentially at low temperatures, they could remain present and effective within cells for generations after a shift to higher, non-inducing temperatures.

Finally, it should be noted that the evolution of resistant phenotypes within our chemostats prior to phage exposure would also explain our observations. *Microcystis* strains are rich in mobile elements (45), and the activity of transposes has been demonstrated to be influenced by environmental conditions (45). Thus, during chemostat acclimation, it is plausible that a population shift toward phage resistance may be occurring; however, this population shift would not be driven by phage because they were absent during the chemostat acclimation period. As phages recognize and bind to receptors on the host organism to establish infections, a mutation that alters the cell envelope or its proteins could prevent recognition (46). This mechanism of viral defense has been shown in many gram-negative bacteria, including *Escherichia coli*, *Synechococcus sp*. WH7803, and *Vibrio cholerae* (47–49). However, the receptor(s) for Ma-LMM01 infection of *M. aeruginosa* NIES-298 remain unknown, and it is likewise unknown how resistance-conferring mutations in these receptors would provide a growth advantage at 19°C (but not 26°C) in our chemostat systems to facilitate takeover of the population prior to phage addition.

### Conclusions

This study provides experimental evidence for a shift from a Ma-LMM01 susceptible to a resistant phenotype in *M. aeruginosa* NIES-298 dependent on host acclimation temperature before infection—an acclimation that provides for increased microcystin concentration in the cells. Going forward, the approach and observation provide a tool to study microcystin and/or other secondary metabolites as a potential anti-viral mechanism. Further studies will need to be conducted to tease apart the mechanism by which resistance is gained under *M. aeruginosa* acclimation at lower temperatures and better describe its environmental impact on *Microcystis* blooms *in situ*.

## Data Availability

The data found within this article are available in the article and in the supplementary material. All code along with corresponding data can be accessed at https://github.com/kennedihambrick/NIES298infection.git.

## SUPPLEMENTAL MATERIAL

Supplemental material spectrum.03379-25-s0001.docx Fig. S1–S14; Tables S1–S5.

ASM does not own the copyrights to Supplemental Material that may be linked to, or accessed through, an article. The authors have granted ASM a non-exclusive, world-wide license to publish the Supplemental Material files. Please contact the corresponding author directly for reuse.

## References

[1] Harke MJ, Gobler CJ. 2015. Daily transcriptome changes reveal the role of nitrogen in controlling microcystin synthesis and nutrient transport in the toxic cyanobacterium, *Microcystis aeruginosa*. BMC Genomics 16:1068. doi:10.1186/s12864-015-2275-9 26673568 PMC4681089

[2] Wilhelm SW, Bullerjahn GS, McKay RML. 2020. The complicated and confusing ecology of *Microcystis* blooms. mBio 11:e00529-20. doi:10.1128/mBio.00529-20 32605981 PMC7327167

[3] Lad A, Breidenbach JD, Su RC, Murray J, Kuang R, Mascarenhas A, Najjar J, Patel S, Hegde P, Youssef M, Breuler J, Kleinhenz AL, Ault AP, Westrick JA, Modyanov NN, Kennedy DJ, Haller ST. 2022. As we drink and breathe: Adverse health effects of microcystins and other harmful algal bloom toxins in the liver, gut, lungs and beyond. Life (Basel) 12:418. doi:10.3390/life12030418 35330169 PMC8950847

[4] Qu J, Shen L, Zhao M, Li W, Jia C, Zhu H, Zhang Q. 2018. Determination of the role of *Microcystis aeruginosa* in toxin generation based on phosphoproteomic profiles. Toxins (Basel) 10:304. doi:10.3390/toxins10070304 30041444 PMC6070999

[5] Lürling M, Van Oosterhout F, Faassen E. 2017. Eutrophication and warming boost cyanobacterial biomass and microcystins. Toxins (Basel) 9:64. doi:10.3390/toxins9020064 28208670 PMC5331443

[6] Li H, Xing P, Chen M, Bian Y, Wu QL. 2011. Short-term bacterial community composition dynamics in response to accumulation and breakdown of *Microcystis* blooms. Water Res 45:1702–1710. doi:10.1016/j.watres.2010.11.011 21185055

[7] Briand E, Escoffier N, Straub C, Sabart M, Quiblier C, Humbert JF. 2009. Spatiotemporal changes in the genetic diversity of a bloom-forming *Microcystis aeruginosa* (cyanobacteria) population. ISME J 3:419–429. doi:10.1038/ismej.2008.121 19092863

[8] Chen F, Xie P, Tang H, Liu H. 2005. Negative effects of *Microcystis* blooms on the crustacean plankton in an enclosure experiment in the subtropical China. J Environ Sci (China) 17:775–781. 16313001

[9] Kardinaal WEA, Tonk L, Janse I, Hol S, Slot P, Huisman J, Visser PM. 2007. Competition for light between toxic and nontoxic strains of the harmful cyanobacterium *Microcystis*. Appl Environ Microbiol 73:2939–2946. doi:10.1128/AEM.02892-06 17337540 PMC1892876

[10] Elbehery AHA, Deng L. 2022. Insights into the global freshwater virome. Front Microbiol 13:953500. doi:10.3389/fmicb.2022.953500 36246212 PMC9554406

[11] Suttle CA. 2000. Cyanophages and their role in the ecology of cyanobacteria, p 563–589. *In* Whitton BA, Potts M (ed), The ecology of cyanobacteria: their diversity in time and space. Kluwer Academic Publisher, Dordrecht.

[12] Yoshida T, Takashima Y, Tomaru Y, Shirai Y, Takao Y, Hiroishi S, Nagasaki K. 2006. Isolation and characterization of a cyanophage infecting the toxic cyanobacterium *Microcystis aeruginosa*. Appl Environ Microbiol 72:1239–1247. doi:10.1128/AEM.72.2.1239-1247.2006 16461672 PMC1392944

[13] Ou T, Li S, Liao X, Zhang Q. 2013. Cultivation and characterization of the MaMV-DC cyanophage that infects bloom-forming cyanobacterium *Microcystis aeruginosa*. Virol Sin 28:266–271. doi:10.1007/s12250-013-3340-7 23990146 PMC8208409

[14] Yoshida T, Nagasaki K, Takashima Y, Shirai Y, Tomaru Y, Takao Y, Sakamoto S, Hiroishi S, Ogata H. 2008. Ma-LMM01 infecting toxic *Microcystis aeruginosa* illuminates diverse cyanophage genome strategies. J Bacteriol 190:1762–1772. doi:10.1128/JB.01534-07 18065537 PMC2258655

[15] Stough JMA, Tang X, Krausfeldt LE, Steffen MM, Gao G, Boyer GL, Wilhelm SW. 2017. Molecular prediction of lytic vs lysogenic states for *Microcystis* phage: metatranscriptomic evidence of lysogeny during large bloom events. PLoS One 12:e0184146. doi:10.1371/journal.pone.0184146 28873456 PMC5584929

[16] Steffen MM, Belisle BS, Watson SB, Boyer GL, Bourbonniere RA, Wilhelm SW. 2015. Metatranscriptomic evidence for co-occurring top-down and bottom-up controls on toxic cyanobacterial communities. Appl Environ Microbiol 81:3268–3276. doi:10.1128/AEM.04101-14 25662977 PMC4393433

[17] Dick GJ, Duhaime MB, Evans JT, Errera RM, Godwin CM, Kharbush JJ, Nitschky HS, Powers MA, Vanderploeg HA, Schmidt KC, Smith DJ, Yancey CE, Zwiers CC, Denef VJ. 2021. The genetic and ecophysiological diversity of *Microcystis*. Environ Microbiol 23:7278–7313. doi:10.1111/1462-2920.15615 34056822

[18] Yang Z, Zhang M, Yu Y, Shi X. 2020. Temperature triggers the annual cycle of *Microcystis*, comparable results from the laboratory and a large shallow lake. Chemosphere 260:127543. doi:10.1016/j.chemosphere.2020.127543 32659542

[19] Yang C, Lin F, Li Q, Li T, Zhao J. 2015. Comparative genomics reveals diversified CRISPR-Cas systems of globally distributed *Microcystis aeruginosa*, a freshwater bloom-forming cyanobacterium. Front Microbiol 6:394. doi:10.3389/fmicb.2015.00394 26029174 PMC4428289

[20] Kuno S, Yoshida T, Kaneko T, Sako Y. 2012. Intricate interactions between the bloom-forming cyanobacterium *Microcystis aeruginosa* and foreign genetic elements, revealed by diversified clustered regularly interspaced short palindromic repeat (CRISPR) signatures. Appl Environ Microbiol 78:5353–5360. doi:10.1128/AEM.00626-12 22636003 PMC3416447

[21] Papoulis SE, Wilhelm SW, Talmy D, Zinser ER, Moran MA. 2021. Nutrient loading and viral memory drive accumulation of restriction modification systems in bloom-forming cyanobacteria. mBio 12:e0087321. doi:10.1128/mBio.00873-21 34060332 PMC8262939

[22] Rinta-Kanto JM, Konopko EA, DeBruyn JM, Bourbonniere RA, Boyer GL, Wilhelm SW. 2009. Lake Erie *Microcystis*: relationship between microcystin production, dynamics of genotypes and environmental parameters in a large lake. Harmful Algae 8:665–673. doi:10.1016/j.hal.2008.12.004

[23] Matteson AR, Loar SN, Bourbonniere RA, Wilhelm SW. 2011. Molecular enumeration of an ecologically important cyanophage in a Laurentian Great Lake. Appl Environ Microbiol 77:6772–6779. doi:10.1128/AEM.05879-11 21841023 PMC3187120

[24] Bishop CT, Anet EF, Gorham PR. 1959. Isolation and identification of the fast-death factor in *Microcystis aeruginosa* NRC-1. Can J Biochem Physiol 37:453–471. doi:10.1139/o59-047 13638864

[25] Tillett D, Dittmann E, Erhard M, von Döhren H, Börner T, Neilan BA. 2000. Structural organization of microcystin biosynthesis in *Microcystis aeruginosa* PCC7806: an integrated peptide-polyketide synthetase system. Chem Biol 7:753–764. doi:10.1016/s1074-5521(00)00021-1 11033079

[26] Wilhelm SW, Boyer GL. 2011. Healthy competition. Nature Clim Change 1:300–301. doi:10.1038/nclimate1202

[27] Peng G, Martin RM, Dearth SP, Sun X, Boyer GL, Campagna SR, Lin S, Wilhelm SW. 2018. Seasonally relevant cool temperatures interact with N chemistry to increase microcystins produced in lab cultures of *Microcystis aeruginosa* NIES-843. Environ Sci Technol 52:4127–4136. doi:10.1021/acs.est.7b06532 29522323

[28] Schuurmans JM, Brinkmann BW, Makower AK, Dittmann E, Huisman J, Matthijs HCP. 2018. Microcystin interferes with defense against high oxidative stress in harmful cyanobacteria. Harmful Algae 78:47–55. doi:10.1016/j.hal.2018.07.008 30196924

[29] Martin RM, Moniruzzaman M, Stark GF, Gann ER, Derminio DS, Wei B, Hellweger FL, Pinto A, Boyer GL, Wilhelm SW. 2020. Episodic decrease in temperature increases mcy gene transcription and cellular microcystin in continuous cultures of *Microcystis aeruginosa* PCC 7806. Front Microbiol 11:601864. doi:10.3389/fmicb.2020.601864 33343544 PMC7744600

[30] Watanabe MM, Ichimura T. 1977. Fresh- and salt-water forms of Spirulina platensis in axenic cultures. Bull Jpn Soc Phycol 25:371–377. https://cir.nii.ac.jp/crid/1570009750474175104.

[31] Boyer GL. 2020. LCMS-SOP determination of microcystins in water samples by high performance liquid chromatography (HPLC) with single quadrupole mass spectrometry (MS). protocols.io. doi:10.17504/protocols.io.bck2iuye

[32] Zhao Y, Zhao Y, Zheng S, Zhao L, Zhang W, Xiao T, Grégori G. 2023. Enhanced resolution of marine viruses with violet side scatter. Cytometry A 103:260–268. doi:10.1002/cyto.a.24674 35929601

[33] Blin K, Shaw S, Augustijn HE, Reitz ZL, Biermann F, Alanjary M, Fetter A, Terlouw BR, Metcalf WW, Helfrich EJN, van Wezel GP, Medema MH, Weber T. 2023. antiSMASH 7.0: new and improved predictions for detection, regulation, chemical structures and visualisation. Nucleic Acids Res 51:W46–W50. doi:10.1093/nar/gkad344 37140036 PMC10320115

[34] Stark GF, Martin RM, Smith LE, Wei B, Hellweger FL, Bullerjahn GS, McKay RML, Boyer GL, Wilhelm SW. 2023. Microcystin aids in cold temperature acclimation: differences between a toxic *Microcystis* wildtype and non-toxic mutant. Harmful Algae 129:102531. doi:10.1016/j.hal.2023.102531 37951605 PMC10640677

[35] Hinson A, Papoulis S, Fiet L, Knight M, Cho P, Szeltner B, Sgouralis I, Talmy D. 2023. A model of algal‐virus population dynamics reveals underlying controls on material transfer. Limnol Oceanogr 68:165–180. doi:10.1002/lno.12256

[36] van der Westhuizen AJ, Eloff JN. 1985. Effect of temperature and light on the toxicity and growth of the blue-green alga *Microcystis aeruginosa* (UV-006). Planta 163:55–59. doi:10.1007/BF00395897 24249268

[37] Birch EW, Ruggero NA, Covert MW. 2012. Determining host metabolic limitations on viral replication via integrated modeling and experimental perturbation. PLoS Comput Biol 8:e1002746. doi:10.1371/journal.pcbi.1002746 23093930 PMC3475664

[38] Wilson WH, Carr NG, Mann NH. 1996. The effect of phosphate status on the kinetics of cyanophage infection in the oceanic cyanobacterium *Synechococcus* sp. WH7803. J Phycol 32:506–516. doi:10.1111/j.0022-3646.1996.00506.x

[39] Meza-Padilla I, McConkey BJ, Nissimov JI. 2024. Structural models predict a significantly higher binding affinity between the NblA protein of cyanophage Ma-LMM01 and the phycocyanin of *Microcystis aeruginosa* NIES-298 compared to the host homolog. Virus Evol 10:veae082. doi:10.1093/ve/veae082 39411151 PMC11477984

[40] Carrieri D, Jurista T, Yazvenko N, Schafer Medina A, Strickland D, Roberts JM. 2021. Overexpression of NblA decreases phycobilisome content and enhances photosynthetic growth of the cyanobacterium *Synechococcus elongatus* PCC 7942. Algal Res 60:102510. doi:10.1016/j.algal.2021.102510

[41] Morimoto D, Kimura S, Sako Y, Yoshida T. 2018. Transcriptome analysis of a bloom-forming cyanobacterium *Microcystis aeruginosa* during Ma-LMM01 phage infection. Front Microbiol 9:2. doi:10.3389/fmicb.2018.00002 29403457 PMC5780444

[42] Zilliges Y, Kehr J-C, Meissner S, Ishida K, Mikkat S, Hagemann M, Kaplan A, Börner T, Dittmann E. 2011. The cyanobacterial hepatotoxin microcystin binds to proteins and increases the fitness of *Microcystis* under oxidative stress conditions. PLoS One 6:e17615. doi:10.1371/journal.pone.0017615 21445264 PMC3060824

[43] Brown S, Mitarai N, Sneppen K. 2022. Protection of bacteriophage-sensitive *Escherichia coli* by lysogens. Proc Natl Acad Sci USA 119:e2106005119. doi:10.1073/pnas.2106005119 35344423 PMC9168506

[44] Howard-Varona C, Hargreaves KR, Abedon ST, Sullivan MB. 2017. Lysogeny in nature: mechanisms, impact and ecology of temperate phages. ISME J 11:1511–1520. doi:10.1038/ismej.2017.16 28291233 PMC5520141

[45] Steffen MM, Dearth SP, Dill BD, Li Z, Larsen KM, Campagna SR, Wilhelm SW. 2014. Nutrients drive transcriptional changes that maintain metabolic homeostasis but alter genome architecture in *Microcystis*. ISME J 8:2080–2092. doi:10.1038/ismej.2014.78 24858783 PMC4184021

[46] Bertozzi Silva J, Storms Z, Sauvageau D. 2016. Host receptors for bacteriophage adsorption. FEMS Microbiol Lett 363:fnw002. doi:10.1093/femsle/fnw002 26755501

[47] Morona R, Krämer C, Henning U. 1985. Bacteriophage receptor area of outer membrane protein OmpA of *Escherichia coli* K-12. J Bacteriol 164:539–543. doi:10.1128/jb.164.2.539-543.1985 3902787 PMC214285

[48] Marston MF, Pierciey FJ, Shepard A, Gearin G, Qi J, Yandava C, Schuster SC, Henn MR, Martiny JBH. 2012. Rapid diversification of coevolving marine *Synechococcus* and a virus. Proc Natl Acad Sci USA 109:4544–4549. doi:10.1073/pnas.1120310109 22388749 PMC3311363

[49] Sun H, Liu M, Fan F, Li Z, Fan Y, Zhang J, Huang Y, Li Z, Li J, Xu J, Kan B. 2021. The type II secretory system mediates phage infection in *Vibrio cholerae*. Front Cell Infect Microbiol 11:662344. doi:10.3389/fcimb.2021.662344 33968805 PMC8101328

