# Supplementary material for: Acclimation temperature influences phage susceptibility in a toxin-producing strain of Microcystis aeruginosa
Source: Microbiol Spectr. 2026 Jun 15;14(7):e03379-25. doi: 10.1128/spectrum.03379-25 (PMC13340012; doi:10.1128/spectrum.03379-25)
Supplement: Supplemental material — Fig. S1–S14; Tables S1–S5. [file spectrum.03379-25-s0001.docx]

Acclimation temperature influences phage susceptibility in a toxin-producing strain of *Microcystis aeruginosa*

Kennedi M. Hambrick,^a^ Laura E. Smith,^a^ Robbie M. Martin,^a^ Bofan Wei,^b^ Raunak Dey,^c^ Gregory L. Boyer,^b^ Joshua S. Weitz,^c,d,e^ David Talmy,^a^ Steven W. Wilhelm,^a^ and Erik R. Zinser,^a^#

^a^ Department of Microbiology, The University of Tennessee, Knoxville, TN 37996 USA

^b^ Department of Chemistry, State University of New York College of Environmental Science and Forestry, Syracuse, NY 13210 USA

^c^ Department of Physics, University of Maryland, College Park, MD, 20742, USA

^d^ Department of Biology, University of Maryland, College Park, MD, 20742, USA

^e^ University of Maryland Institute for Health Computing, North Bethesda, MD, 20852, USA

Running Head: *Microcystis aeruginosa* NIES-298 phage susceptibility and resistance

#Address correspondence to Erik R. Zinser Tel: +1 865-974-9283; Fax: +1 865-974-4007; Email: ezinser@utk.edu

**Supplementary Materials**

**Supplemental Figure 1:** *M. aeruginosa* Temperature throughout Chemostat Acclimation. Temperature of chemostat acclimated *M. aeruginosa* NIES-298 was logged every 15 minutes *via* HOBO TidbiT Temperature Logger (Onset Computer Corporation) starting at day 10 of chemostat incubation until the end of the acclimation period. The blue shading represents time period in which the incubator was lowered to 19° C. The vertical dashed lines indicate times at which samples were taken from chemostats for batch culture infectivity experiments.

**Supplemental Table 1:** One way ANOVA on 26° C and 19° C acclimated host baseline abundances. P-values indicate significant difference in baseline abundances within and between acclimation temperature treatments. No significant difference in abundances were found. Brown-Forsythe and Welch ANOVA multiple comparisons with Dunnett’s correction was utilized.

|  | T_23_ | T_24_ | T_25_ | T_28_ | T_29_ | T_30_ | T_32_ | T_35_ | T_42_ | T_43_ | T_44_ | T_45_ |
| --- | --- | --- | --- | --- | --- | --- | --- | --- | --- | --- | --- | --- |
| T_23_ | **-** | >0.9999 | >0.9999 | >0.9999 | >0.9999 | 0.998 | 0.9936 | >0.9999 | >0.9999 | >0.9999 | >0.9999 | >0.9999 |
| T_24_ | **-** | **-** | >0.9999 | >0.9999 | >0.9999 | >0.9999 | 0.9998 | >0.9999 | >0.9999 | >0.9999 | >0.9999 | >0.9999 |
| T_25_ | **-** | **-** | **-** | >0.9999 | >0.9999 | >0.9999 | >0.9999 | >0.9999 | >0.9999 | >0.9999 | >0.9999 | >0.9999 |
| T_28_ | **-** | **-** | **-** | **-** | >0.9999 | 0.9966 | 0.9978 | >0.9999 | >0.9999 | >0.9999 | >0.9999 | >0.9999 |
| T_29_ | **-** | **-** | **-** | **-** | **-** | >0.9999 | >0.9999 | >0.9999 | 0.9959 | >0.9999 | >0.9999 | 0.9926 |
| T_30_ | **-** | **-** | **-** | **-** | **-** | **-** | >0.9999 | >0.9999 | 0.965 | 0.9975 | 0.9973 | 0.903 |
| T_32_ | **-** | **-** | **-** | **-** | **-** | **-** | **-** | >0.9999 | 0.9487 | 0.9755 | 0.9886 | 0.8916 |
| T_35_ | **-** | **-** | **-** | **-** | **-** | **-** | **-** | **-** | 0.9994 | >0.9999 | >0.9999 | 0.9902 |
| T_42_ | **-** | **-** | **-** | **-** | **-** | **-** | **-** | **-** | **-** | >0.9999 | 0.9984 | >0.9999 |
| T_43_ | **-** | **-** | **-** | **-** | **-** | **-** | **-** | **-** | **-** | **-** | >0.9999 | 0.9998 |
| T_44_ | **-** | **-** | **-** | **-** | **-** | **-** | **-** | **-** | **-** | **-** | **-** | 0.9767 |
| T_45_ | **-** | **-** | **-** | **-** | **-** | **-** | **-** | **-** | **-** | **-** | **-** | **-** |

**Supplemental Table 2:** One way ANOVA on 26° C and 19° C acclimated host baseline intracellular microcystin concentrations. P-values indicate significant difference in baseline intracellular microcystin concentrations within and between acclimation temperature treatments. Brown-Forsythe and Welch ANOVA multiple comparisons with Dunnett’s correction was utilized.

|  | T_23_ | T_24_ | T_25_ | T_28_ | T_29_ | T_30_ | T_32_ | T_35_ | T_42_ | T_43_ | T_44_ | T_45_ |
| --- | --- | --- | --- | --- | --- | --- | --- | --- | --- | --- | --- | --- |
| T_23_ | **-** | 0.9844 | >0.9999 | >0.9999 | 0.8002 | 0.9728 | >0.9999 | 0.9816 | 0.3576 | 0.3241 | 0.6894 | 0.1129 |
| T_24_ | **-** | **-** | 0.8196 | >0.9999 | 0.924 | 0.9992 | 0.9995 | >0.9999 | 0.3746 | 0.3364 | 0.7284 | 0.0107 |
| T_25_ | **-** | **-** | **-** | >0.9999 | 0.7547 | 0.9732 | >0.9999 | 0.3653 | 0.337 | 0.3127 | 0.6891 | 0.0438 |
| T_28_ | **-** | **-** | **-** | **-** | 0.9697 | 0.9988 | >0.9999 | >0.9999 | 0.3096 | 0.2259 | 0.7195 | 0.2506 |
| T_29_ | **-** | **-** | **-** | **-** | **-** | >0.9999 | 0.9306 | 0.8629 | 0.3975 | 0.2701 | 0.8271 | 0.2232 |
| T_30_ | **-** | **-** | **-** | **-** | **-** | **-** | 0.9969 | 0.9962 | 0.535 | 0.3502 | 0.8389 | 0.3632 |
| T_32_ | **-** | **-** | **-** | **-** | **-** | **-** | **-** | >0.9999 | 0.2623 | 0.1991 | 0.7065 | 0.2031 |
| T_35_ | **-** | **-** | **-** | **-** | **-** | **-** | **-** | **-** | 0.356 | 0.3253 | 0.7153 | 0.0433 |
| T_42_ | **-** | **-** | **-** | **-** | **-** | **-** | **-** | **-** | **-** | 0.9406 | >0.9999 | 0.7894 |
| T_43_ | **-** | **-** | **-** | **-** | **-** | **-** | **-** | **-** | **-** | **-** | 0.9993 | >0.9999 |
| T_44_ | **-** | **-** | **-** | **-** | **-** | **-** | **-** | **-** | **-** | **-** | **-** | 0.9997 |
| T_45_ | **-** | **-** | **-** | **-** | **-** | **-** | **-** | **-** | **-** | **-** | **-** | **-** |

**Supplemental Table 3**: One way ANOVA on 26° C acclimated host infection experiment. P-values indicate significant difference in 26° C acclimated host cell concentration in hosts (i) uninfected vs. infected at 26° C, (ii) uninfected at 26° C vs. uninfected at 19° C , (iii) uninfected vs. infected at 19° C, and (iv) infected at 26° C vs. infected at 19° C. Brown-Forsythe and Welch ANOVA multiple comparisons with Dunnett’s correction was utilized.

| Treatment | Adjusted P-Value |
| --- | --- |
| 26° C uninfected vs. 26° C infected | T_0_ = 0.9756  T_1_ = 0.7201  T_2_ = 0.0519  T_3_ <0.0001  T_4_ <0.0001  T_5_ <0.0001  T_6_ <0.0001  T_7_ = 0.0011  T_8_ = 0.0112 |
| 26° C uninfected vs. 19° C uninfected | T_0_ >0.9999  T_1_ = 0.0573  T_2_ = 0.1392  T_3_ = 0.0299  T_4_ = 0.1304  T_5_ = 0.6212  T_6_ = 0.9552  T_7_ >0.9999  T_8_ = 0.9995 |
| 19° C uninfected vs. 19° C infected | T_0_ >0.9999  T_1_ = 0.9782  T_2_ = 0.9728  T_3_ = 0.2415  T_4_ = 0.0689  T_5_ = 0.029  T_6_ = 0.017  T_7_ = 0.0063  T_8_ = 0.0017 |
| 26° C infected vs. 19° C infected | T_0_ = 0.972  T_1_ = 0.6147  T_2_ = 0.9702  T_3_ = 0.0019  T_4_ = 0.0057  T_5_ = 0.0744  T_6_ = 0.6316  T_7_ = 0.8705  T_8_ = 0.7726 |

**Supplemental Table 4**: One way ANOVA on 19° C acclimated host infection experiment. P-values indicate significant difference in 19° C acclimated host cell concentration in hosts (i) uninfected vs. infected at 26° C, (ii) uninfected at 26° C vs. uninfected at 19° C, (iii) uninfected vs infected at 19° C, and (iv) infected at 26° C vs. infected at 19° C. Brown-Forsythe and Welch ANOVA multiple comparisons with Dunnett’s correction was utilized.

| Treatment | Adjusted P-Value |
| --- | --- |
| 26° C uninfected vs. 26° C infected | T_0_ = 0.9997  T_1_ = 0.5357  T_2_ = 0.0003  T_3_ <0.0001  T_4_ <0.0001  T_5_ = 0.0015  T_6_ = 0.0072  T_7_ = 0.006  T_8_ = 0.0581 |
| 26° C uninfected vs. 19° C uninfected | T_0_ >0.9999  T_1_ = 0.0023  T_2_ =0.0004  T_3_ >0.9999  T_4_ = 0.6849  T_5_ = 0.3439  T_6_ = 0.5227  T_7_ = 0.9865  T_8_ >0.9999 |
| 19° C uninfected vs. 19° C infected | T_0_ >0.9999  T_1_ = 0.9907  T_2_ = 0.1879  T_3_ <0.0001  T_4_ <0.0001  T_5_ <0.0001  T_6_ <0.0001  T_7_ = 0.003  T_8_ = 0.1043 |
| 26° C infected vs. 19° C infected | T_0_ >0.9999  T_1_ <0.0001  T_2_ >0.9999  T_3_ <0.0001  T_4_ = 0.6896  T_5_ = 0.1739  T_6_ = 0.0196  T_7_ = 0.0358  T_8_ = 0.0189 |

**Supplemental Figure 2:** Uninfected cultures from Infection experiment of *Microcystis aeruginosa* NIES-298. Log transformed average cell concentrations during batch culture infection experiment of algal host acclimated in chemostat at (A) 26° C and (B) 19 °C. The x-axis is incubation time in units of days. The left y-axis represents log transformed *M. aeruginosa* NIES-298 cell concentration. A large stimulation from lysate materials that were not viral was not shown in either *M. aeruginosa* acclimated at 26° C or 19° C and supplemented with 0.02μm filter sterilized lysate. Host without cyanophage infection at 26° C is represented by the black closed circles, host without cyanophage infection at 19° C is represented by the black closed squares, host without cyanophage infection but with 0.02μm filter sterilized Ma-LMM01 lysate addition at 26° C is represented by red closed circles, and host without cyanophage infection but with 0.02μm filter sterilized Ma-LMM01 lysate addition at 19° C is represented by blue closed squares. Error bars indicate mean $\pm$ SD.

**
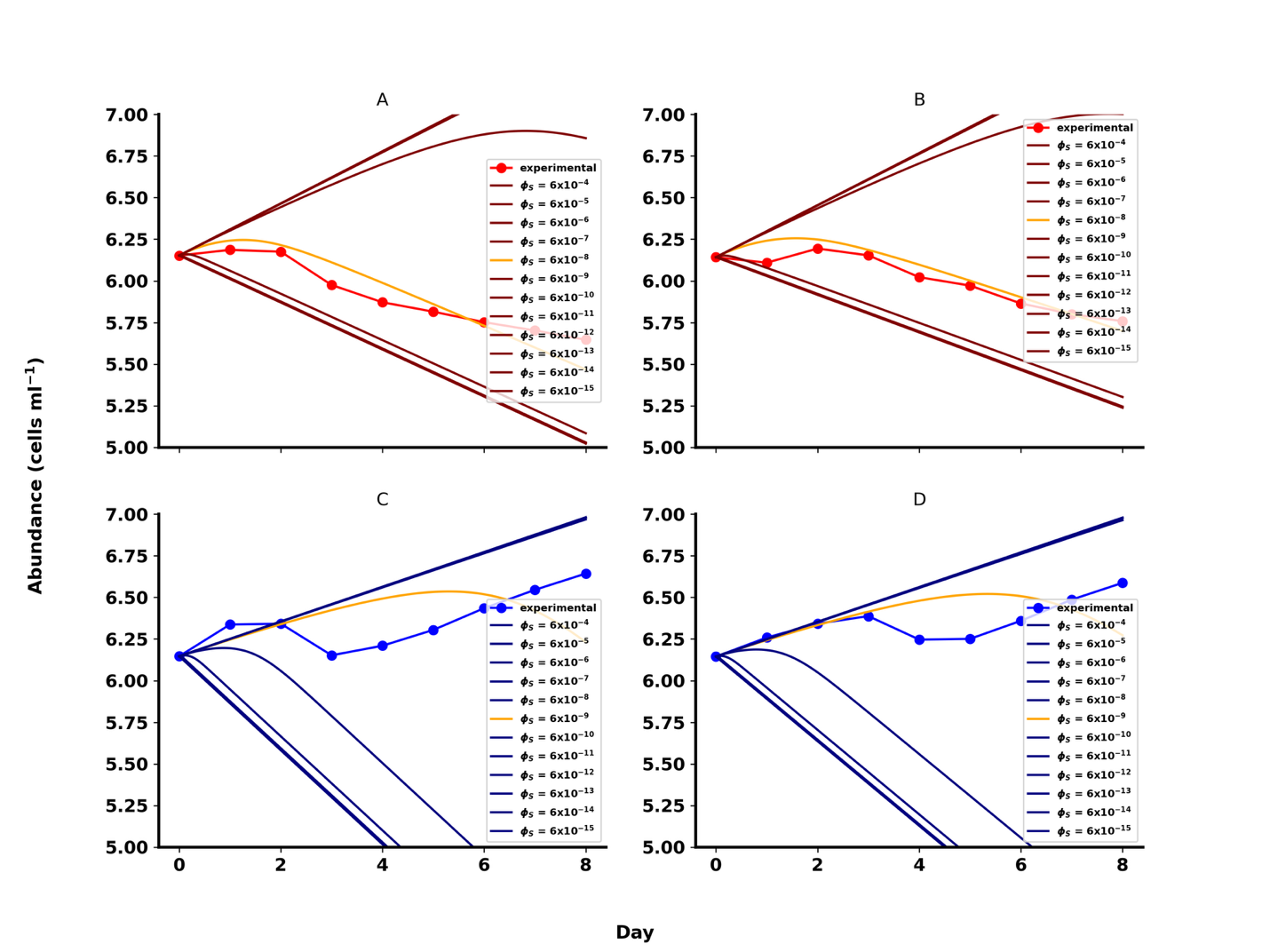
**

**Supplemental Figure 3**: Sensitivity analysis to the parameter ‘$\varphi_{S}$’ in a *M. aeruginosa* NIES-298 Lytic Population Model. A) 26° C acclimated algal hosts infected with cyanophage at 26° C, (B) 26° C acclimated algal hosts infected with cyanophage at 19° C, (C) 19° C acclimated algal hosts infected with cyanophage at 26° C, and (D) 19° C acclimated algal hosts infected with cyanophage at 19° C. The y-axis is log transformed algal cell concentration in (cells mL^-1^) and includes both ‘susceptible’ and ‘infected’ populations. The model solution with the highest likelihood value is shown in orange for each subplot.

**
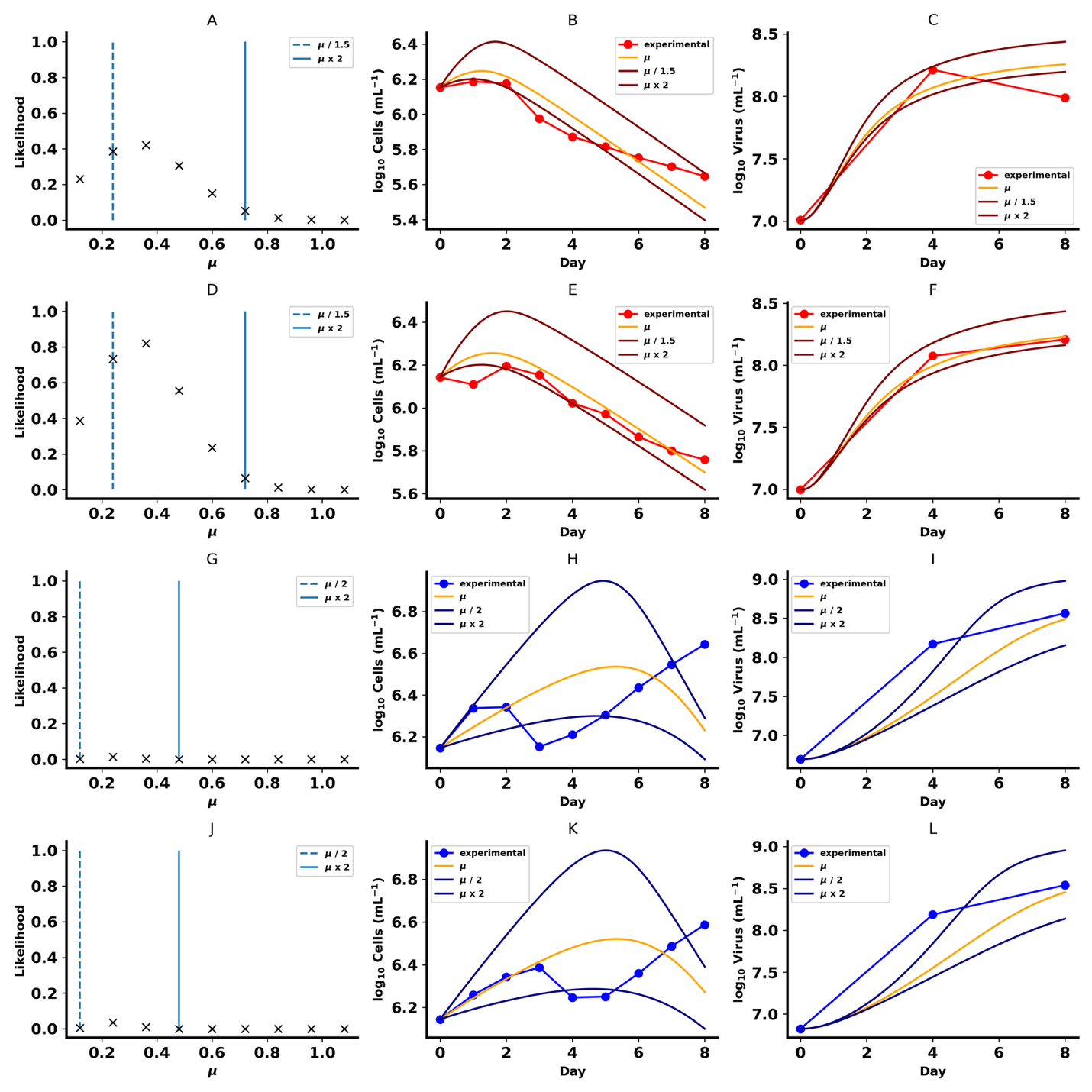
**

**Supplemental Figure 4**: Sensitivity analysis to the key parameter ‘$\mu$’ in the *M. aeruginosa* NIES-298 Lytic Population Model. Effect of $\mu$ on the (A) likelihood for the 26° C acclimated/26° C infected host, (B) log transformed algal host abundance for the 26° C acclimated/26° C infected host, (C) log transformed viral abundance for the 26° C acclimated/26° C infected host, (D) likelihood for the 26° C acclimated/19° C infected host, (E) log transformed algal host abundance for the 26° C acclimated/19° C infected host, (F) log transformed viral abundance for the 26° C acclimated/19° C infected host, (G) likelihood for the 19° C acclimated/26° C infected host, (H) log transformed algal host abundance for the 19° C acclimated/26° C infected host, (I) log transformed viral abundance for the 19° C acclimated/26° C infected host, (J) likelihood for the 19° C acclimated/19° C infected host, (K) log transformed algal host abundance for the 19° C acclimated/19° C infected host, and (L) log transformed viral abundance for the 19° C acclimated/19° C infected host. Dashed lines (A,D) represent $\mu$/1.5 and (G,J) $\mu$/2. Solid lines (A,D,G,J) represent $\mu$x2. Closed circles represents experimental data, solid gold line represents model with greatest likelihood, solid blue/red line represents models with lower likelihood (B,C,E,F,H,I,K,L).


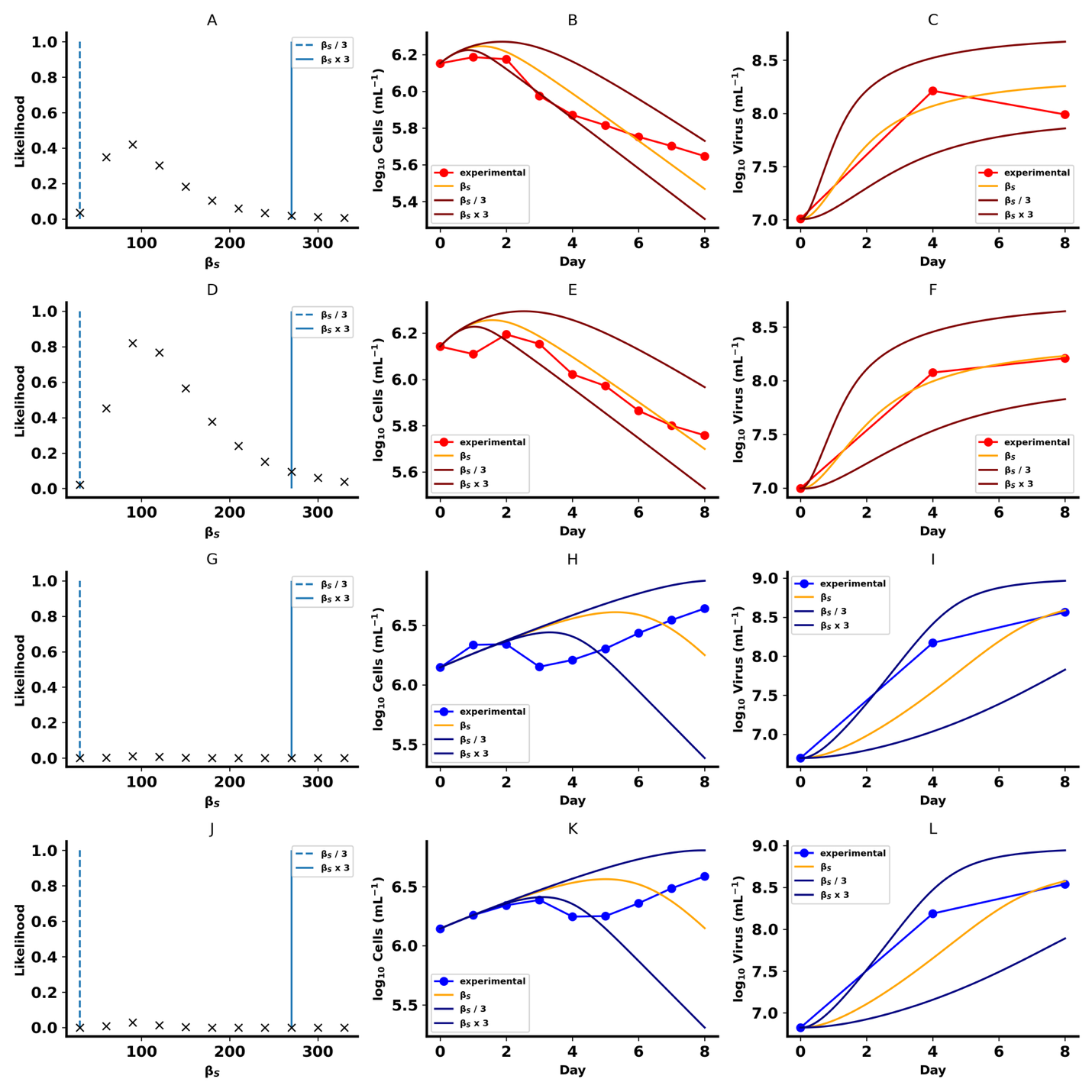


**Supplemental Figure 5**: Sensitivity analysis to the parameter ‘$\beta_{s}$’ in the *M. aeruginosa* NIES-298 Lytic Population Model. Effect of $\beta_{s}$ on the (A) likelihood for the 26° C acclimated/26° C infected host, (B) log transformed algal host abundance for the 26° C acclimated/26° C infected host, (C) log transformed viral abundance for the 26° C acclimated/26° C infected host, (D) likelihood for the 26° C acclimated/19° C infected host, (E) log transformed algal host abundance for the 26° C acclimated/19° C infected host, (F) log transformed viral abundance for the 26° C acclimated/19° C infected host, (G) likelihood for the 19° C acclimated/26° C infected host, (H) log transformed algal host abundance for the 19° C acclimated/26° C infected host, (I) log transformed viral abundance for the 19° C acclimated/26° C infected host, (J) likelihood for the 19° C acclimated/19° C infected host, (K) log transformed algal host abundance for the 19° C acclimated/19° C infected host, and (L) log transformed viral abundance for the 19° C acclimated/19° C infected host. Dashed lines (A,D,G,J) represent $\beta_{s}$/3. Solid lines (A,D,G,J) represent $\beta_{s}$x3. Closed circles represents experimental data, solid gold line represents model with greatest likelihood, solid blue/red line represents models with lower likelihood (B,C,E,F,H,I,K,L).

**
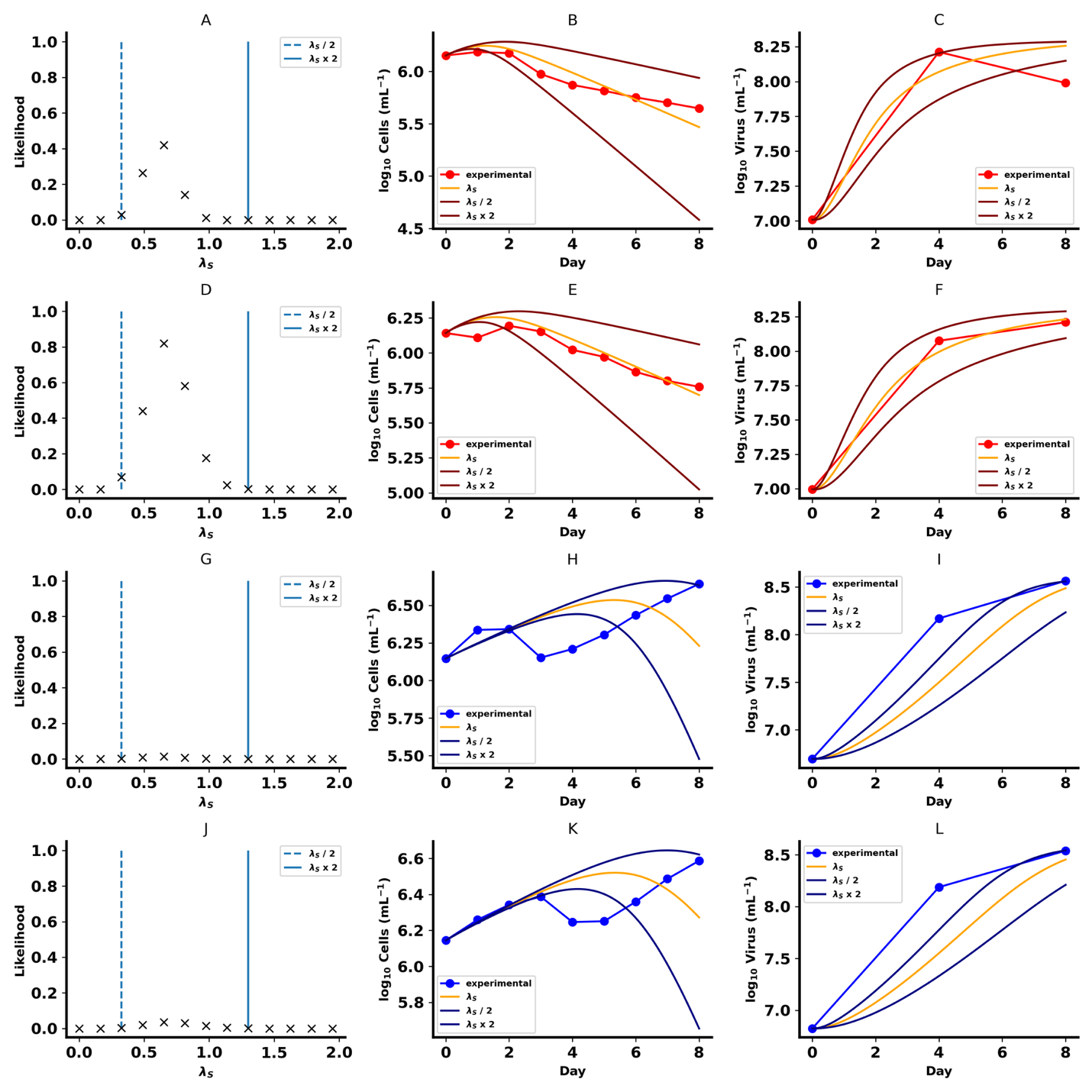
**

**Supplemental Figure 6**: Sensitivity analysis to the parameter ‘$\lambda_{S}$’ in the *M. aeruginosa* NIES-298 Lytic Population Model. Effect of $\lambda_{S}$ on the (A) likelihood for the 26° C acclimated/26° C infected host, (B) log transformed algal host abundance for the 26° C acclimated/26° C infected host, (C) log transformed viral abundance for the 26° C acclimated/26° C infected host, (D) likelihood for the 26° C acclimated/19° C infected host, (E) log transformed algal host abundance for the 26° C acclimated/19° C infected host, (F) log transformed viral abundance for the 26° C acclimated/19° C infected host, (G) likelihood for the 19° C acclimated/26° C infected host, (H) log transformed algal host abundance for the 19° C acclimated/26° C infected host, (I) log transformed viral abundance for the 19° C acclimated/26° C infected host, (J) likelihood for the 19° C acclimated/19° C infected host, (K) log transformed algal host abundance for the 19° C acclimated/19° C infected host, and (L) log transformed viral abundance for the 19° C acclimated/19° C infected host. Dashed lines (A,D,G,J) represent $\lambda_{s}$/2. Solid lines (A,D,G,J) represent $\lambda_{s}$x2. Closed circles represents experimental data, solid gold line represents model with greatest likelihood, solid blue/red line represents models with lower likelihood (B,C,E,F,H,I,K,L).


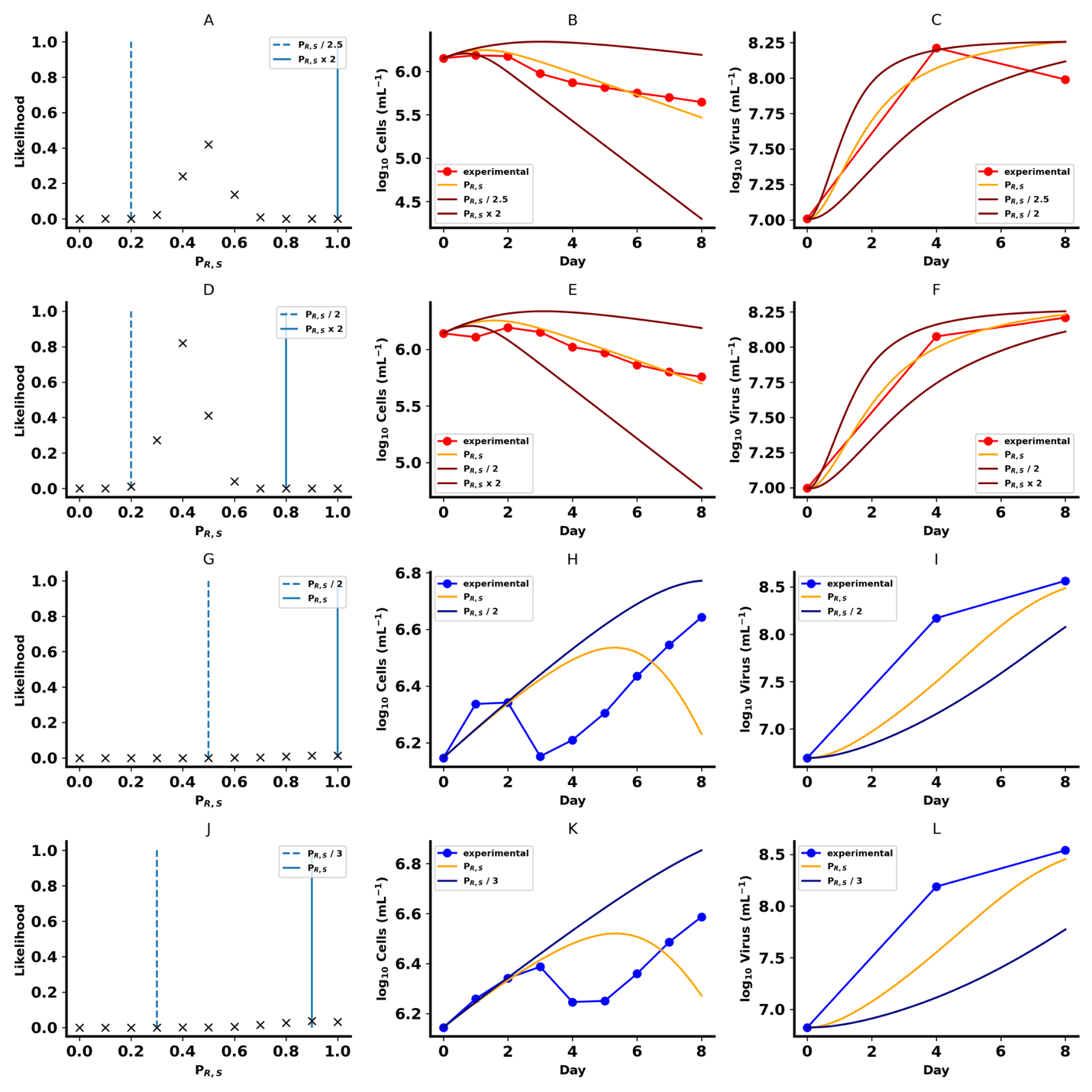


**Supplemental Figure 7**: Sensitivity analysis to the parameter ‘$P_{R,S}$’ in the *M. aeruginosa* NIES-298 Lytic Population Model. Effect of $P_{R,S}$ on the (A) likelihood for the 26° C acclimated/26° C infected host, (B) log transformed algal host abundance for the 26° C acclimated/26° C infected host, (C) log transformed viral abundance for the 26° C acclimated/26° C infected host, (D) likelihood for the 26° C acclimated/19° C infected host, (E) log transformed algal host abundance for the 26° C acclimated/19° C infected host, (F) log transformed viral abundance for the 26° C acclimated/19° C infected host, (G) likelihood for the 19° C acclimated/26° C infected host, (H) log transformed algal host abundance for the 19° C acclimated/26° C infected host, (I) log transformed viral abundance for the 19° C acclimated/26° C infected host, (J) likelihood for the 19° C acclimated/19° C infected host, (K) log transformed algal host abundance for the 19° C acclimated/19° C infected host, and (L) log transformed viral abundance for the 19° C acclimated/19° C infected host. Dashed lines (A) represent $P_{R,S}$/2.5, (D,G) $P_{R,S}$/2, and (J) $P_{R,S}$/3. Solid lines (A,D) represent $P_{R,S}$x2 and (G,J) $P_{R,S}$. Closed circles represents experimental data, solid gold line represents model with greatest likelihood, solid blue/red line represents models with lower likelihood (B,C,E,F,H,I,K,L).


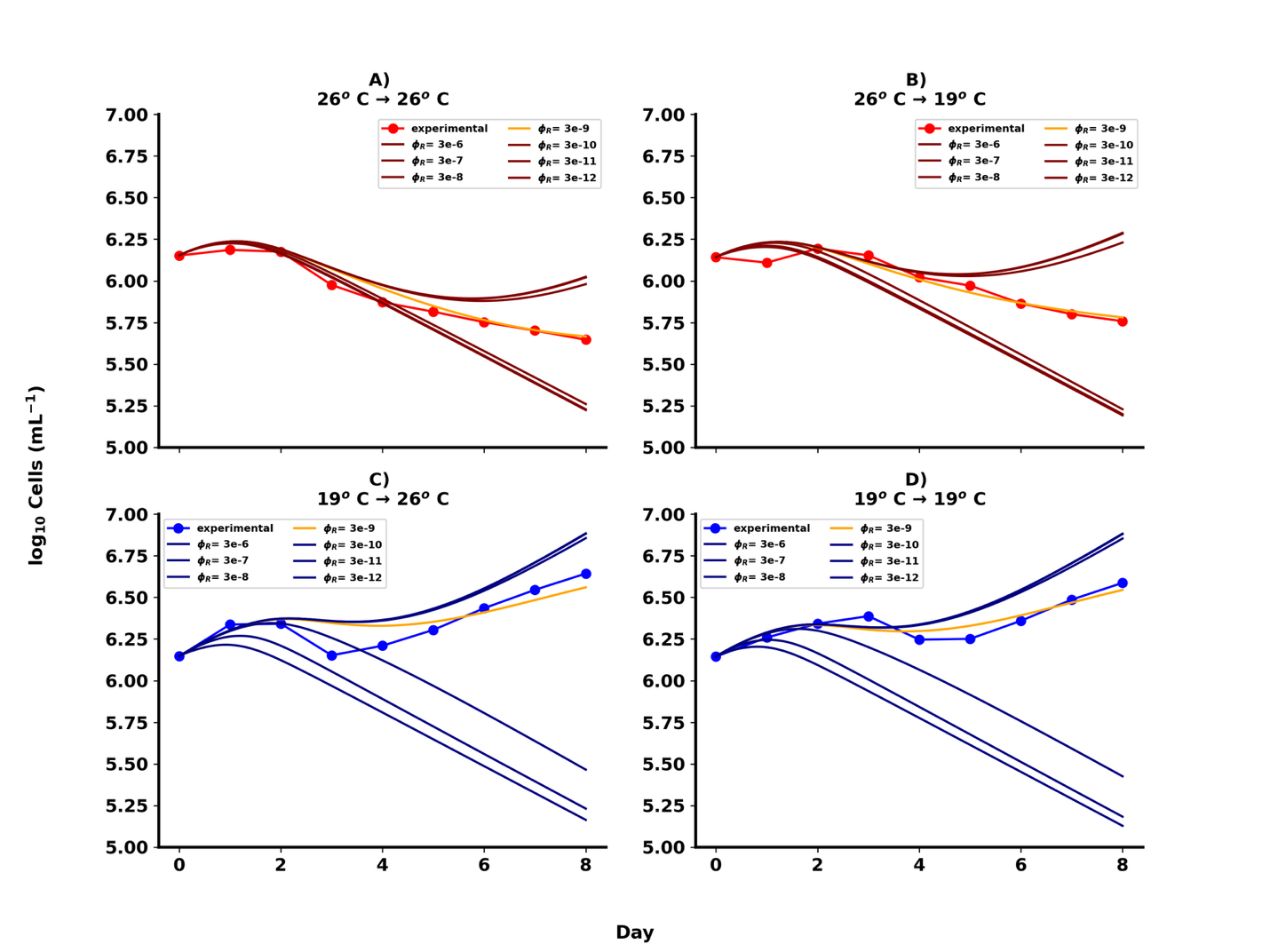


**Supplemental Figure 8**: Sensitivity analysis to the parameter ‘$\varphi_{R}$’ in the *M. aeruginosa* NIES-298 Resistant Subpopulation Model for (A) 26° C acclimated algal hosts infected with cyanophage at 26° C, (B) 26° C acclimated algal hosts infected with cyanophage at 19° C, (C) 19° C acclimated algal hosts infected with cyanophage at 26° C, and (D) 19° C acclimated algal hosts infected with cyanophage at 19° C. The y-axis is the log transformed algal cell concentration in (cells mL^-1^) and includes both ‘susceptible’, ’resistant’, and ‘infected’ populations. The model with the highest likelihood value is shown in orange for each subplot.

**
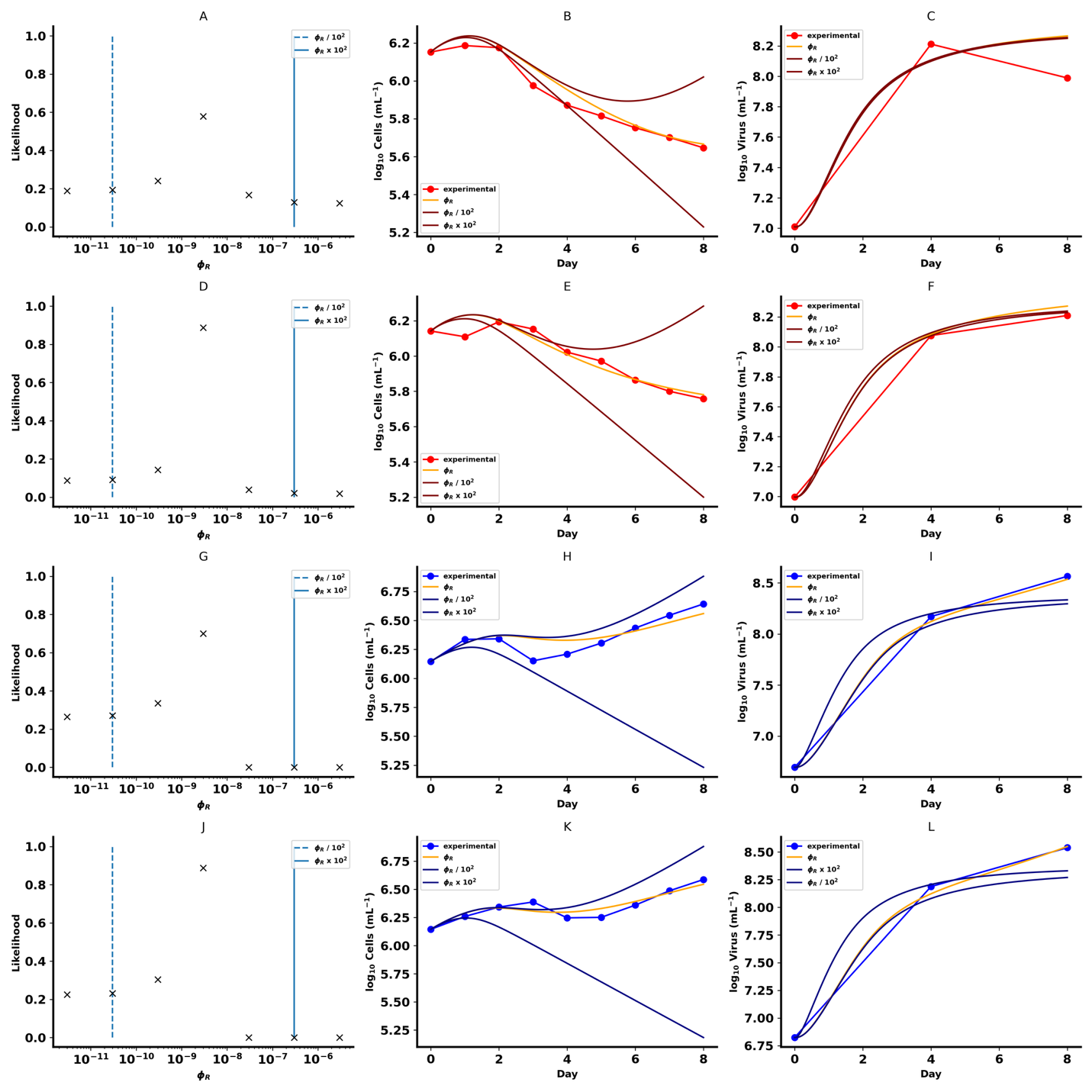
**

**Supplemental Figure 9**: Sensitivity analysis to the parameter ‘$\varphi_{R}$’ in the *M. aeruginosa* NIES-298 Resistant Subpopulation Model. Effect of $\varphi_{R}$ on the (A) likelihood for the 26° C acclimated/26° C infected host, (B) log transformed algal host abundance for the 26° C acclimated/26° C infected host, (C) log transformed viral abundance for the 26° C acclimated/26° C infected host, (D) likelihood for the 26° C acclimated/19° C infected host, (E) log transformed algal host abundance for the 26° C acclimated/19° C infected host, (F) log transformed viral abundance for the 26° C acclimated/19° C infected host, (G) likelihood for the 19° C acclimated/26° C infected host, (H) log transformed algal host abundance for the 19° C acclimated/26° C infected host, (I) log transformed viral abundance for the 19° C acclimated/26° C infected host, (J) likelihood for the 19° C acclimated/19° C infected host, (K) log transformed algal host abundance for the 19° C acclimated/19° C infected host, and (L) log transformed viral abundance for the 19° C acclimated/19° C infected host. Dashed lines (A,D,G,J) represent $\varphi_{R}$/10^2^ . Solid lines (A,D,G,J) represent $\varphi_{R}$x10^2^. Closed circles represents experimental data, solid gold line represents model with greatest likelihood, solid blue/red line represents models with lower likelihood (B,C,E,F,H,I,K,L).


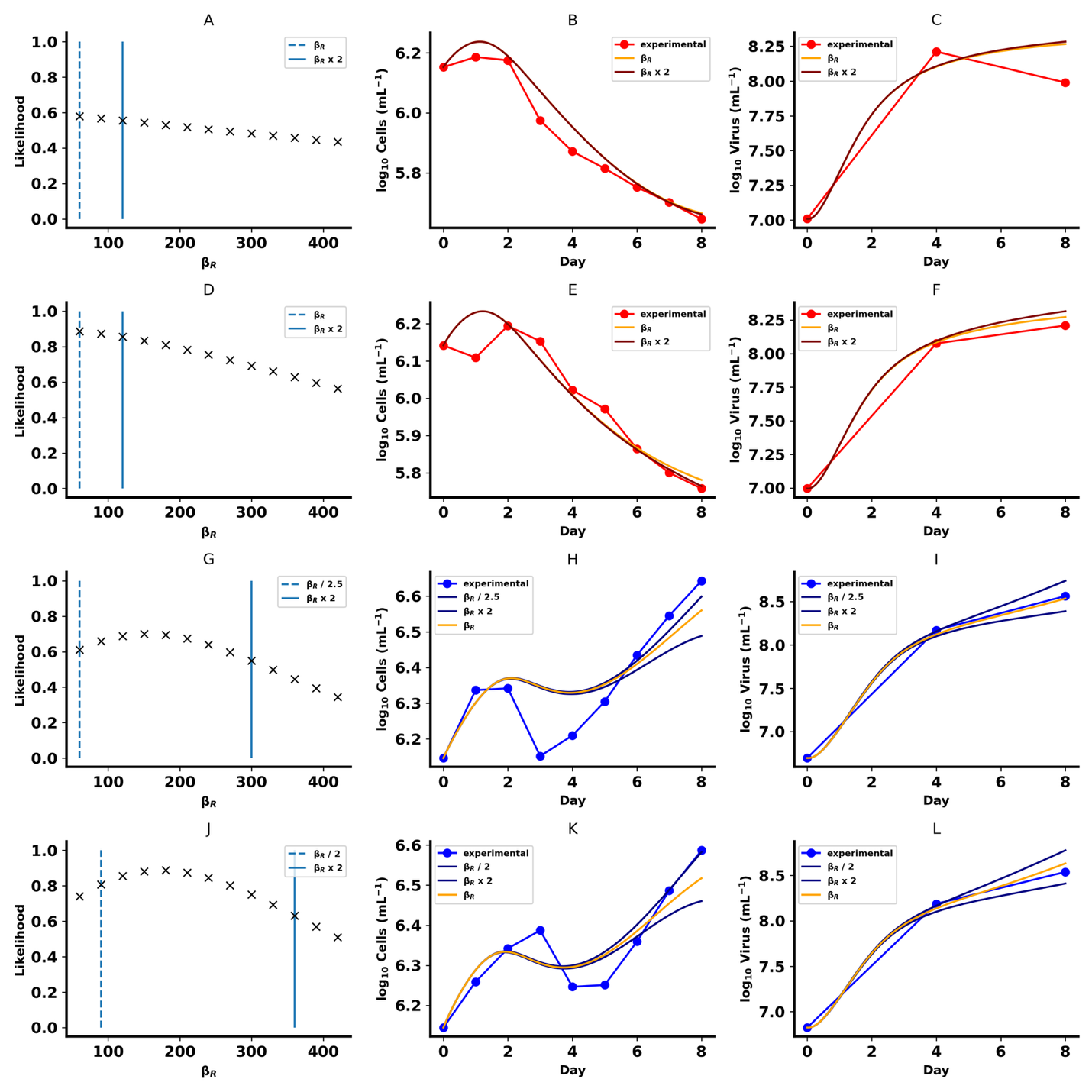


**Supplemental Figure 10**: Sensitivity analysis to the parameter ‘$\beta_{R}$’ in the *M. aeruginosa* NIES-298 Resistant Subpopulation Model. Effect of $\beta_{R}$ on the (A) likelihood for the 26° C acclimated/26° C infected host, (B) log transformed algal host abundance for the 26° C acclimated/26° C infected host, (C) log transformed viral abundance for the 26° C acclimated/26° C infected host, (D) likelihood for the 26° C acclimated/19° C infected host, (E) log transformed algal host abundance for the 26° C acclimated/19° C infected host, (F) log transformed viral abundance for the 26° C acclimated/19° C infected host, (G) likelihood for the 19° C acclimated/26° C infected host, (H) log transformed algal host abundance for the 19° C acclimated/26° C infected host, (I) log transformed viral abundance for the 19° C acclimated/26° C infected host, (J) likelihood for the 19° C acclimated/19° C infected host, (K) log transformed algal host abundance for the 19° C acclimated/19° C infected host, and (L) log transformed viral abundance for the 19° C acclimated/19° C infected host. Dashed lines (A,D) represent $\beta_{R}$, (G) $\beta_{R}$/2.5, and (J) $\beta_{R}$/2 . Solid lines (A,D,G,J) represent $\beta_{R}$x2. Closed circles represents experimental data, solid gold line represents model with greatest likelihood, solid blue/red line represents models with lower likelihood (B,C,E,F,H,I,K,L).


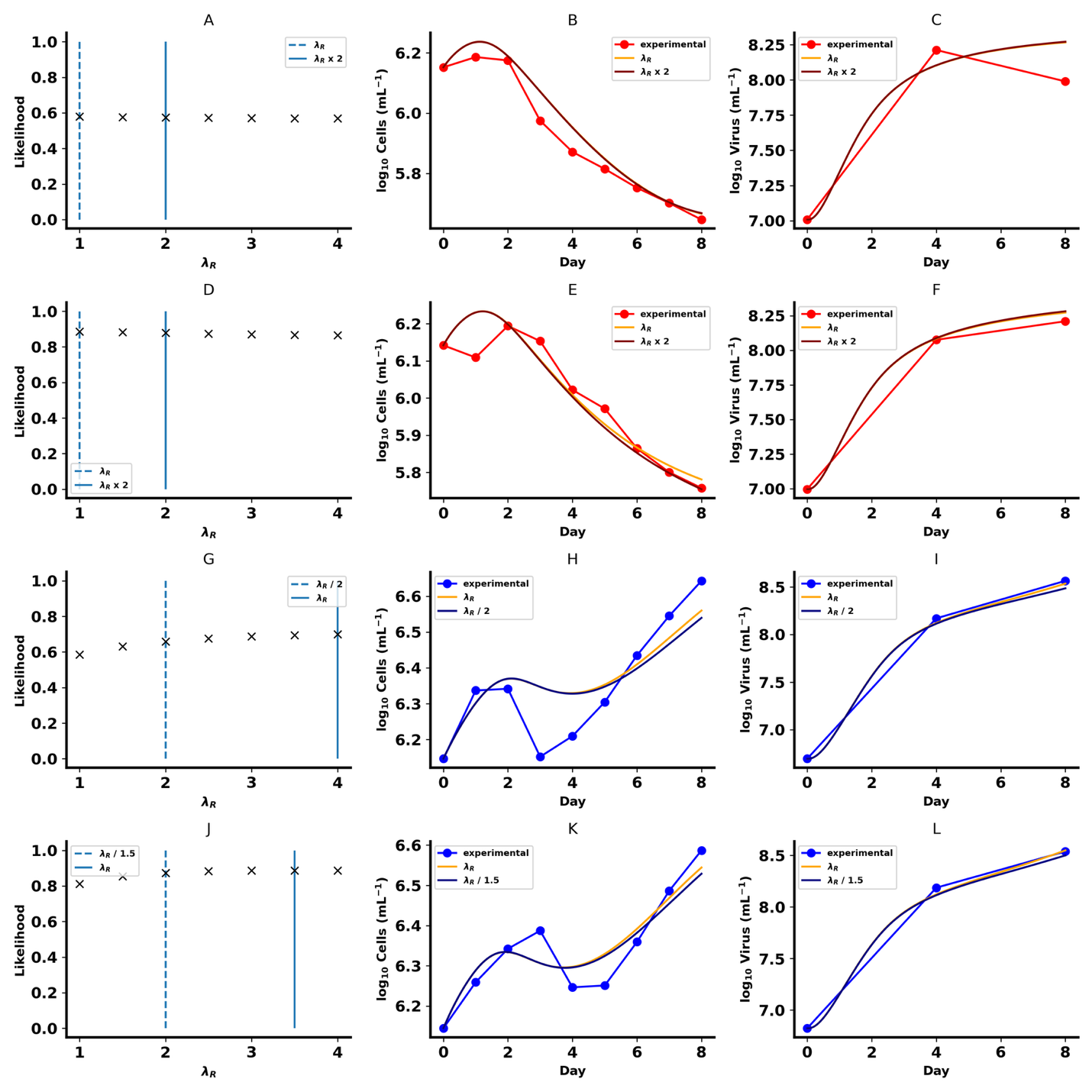


**Supplemental Figure 11**: Sensitivity analysis to the parameter ‘$\lambda_{R}$’ in the *M. aeruginosa* NIES-298 Resistant Subpopulation Model. Effect of $\lambda_{R}$ on the (A) likelihood for the 26° C acclimated/26° C infected host, (B) log transformed algal host abundance for the 26° C acclimated/26° C infected host, (C) log transformed viral abundance for the 26° C acclimated/26° C infected host, (D) likelihood for the 26° C acclimated/19° C infected host, (E) log transformed algal host abundance for the 26° C acclimated/19° C infected host, (F) log transformed viral abundance for the 26° C acclimated/19° C infected host, (G) likelihood for the 19° C acclimated/26° C infected host, (H) log transformed algal host abundance for the 19° C acclimated/26° C infected host, (I) log transformed viral abundance for the 19° C acclimated/26° C infected host, (J) likelihood for the 19° C acclimated/19° C infected host, (K) log transformed algal host abundance for the 19° C acclimated/19° C infected host, and (L) log transformed viral abundance for the 19° C acclimated/19° C infected host. Dashed lines (A,D) represent $\lambda_{R}$, (G) $\lambda_{R}$/2, and (j) $\lambda_{R}$/1.5. Solid lines (A,D) represent $\lambda_{R}$x2 and (G,J) $\lambda_{R}$. Closed circles represents experimental data, solid gold line represents model with greatest likelihood, solid blue/red line represents models with lower likelihood (B,C,E,F,H,I,K,L).


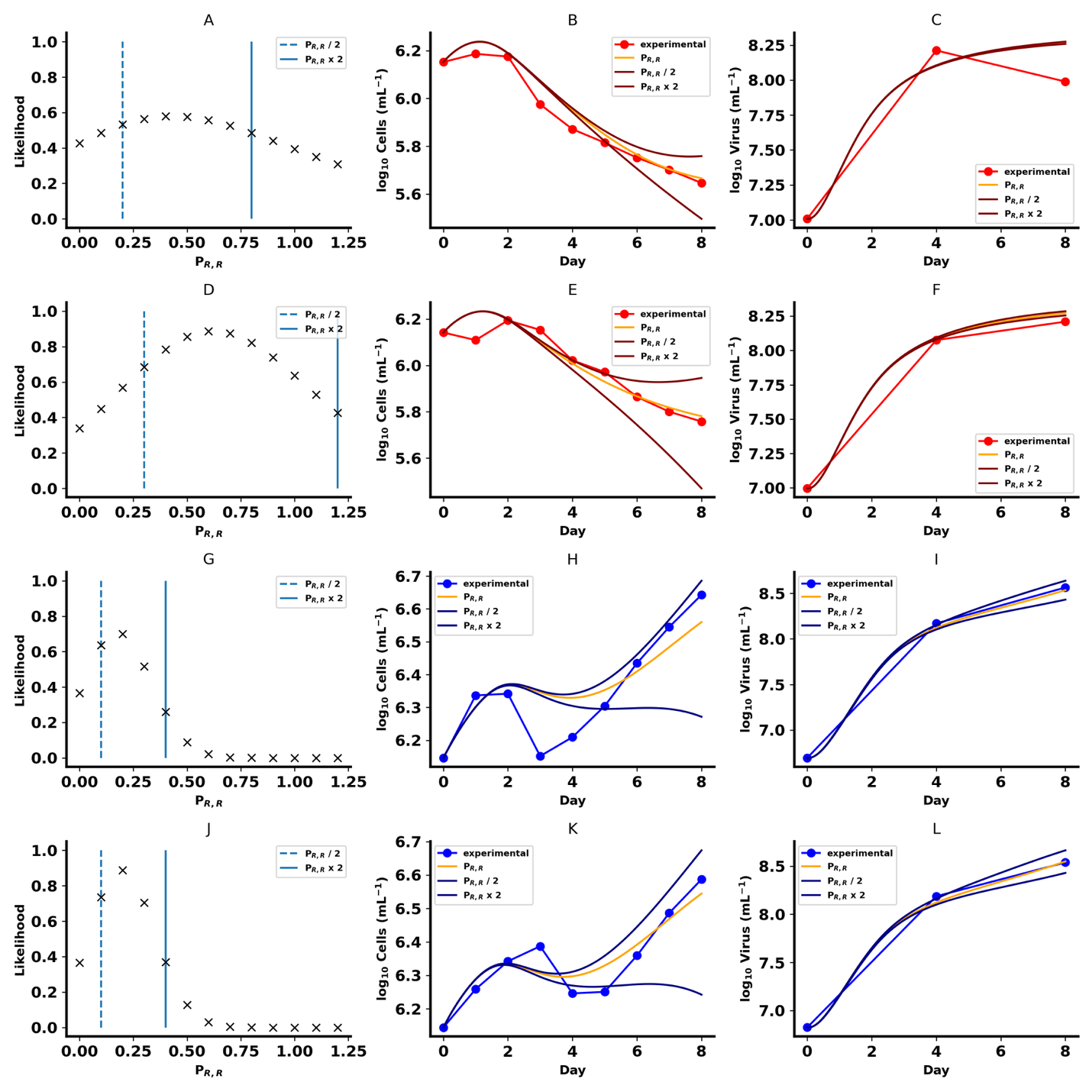


**Supplemental Figure 12**: Sensitivity analysis to the parameter ‘$P_{R,R}$’ in the *M. aeruginosa* NIES-298 Resistant Subpopulation Model. Effect of $P_{R,R}$ on the (A) likelihood for the 26° C acclimated/26° C infected host, (B) log transformed algal host abundance for the 26° C acclimated/26° C infected host, (C) log transformed viral abundance for the 26° C acclimated/26° C infected host, (D) likelihood for the 26° C acclimated/19° C infected host, (E) log transformed algal host abundance for the 26° C acclimated/19° C infected host, (F) log transformed viral abundance for the 26° C acclimated/19° C infected host, (G) likelihood for the 19° C acclimated/26° C infected host, (H) log transformed algal host abundance for the 19° C acclimated/26° C infected host, (I) log transformed viral abundance for the 19° C acclimated/26° C infected host, (J) likelihood for the 19° C acclimated/19° C infected host, (K) log transformed algal host abundance for the 19° C acclimated/19° C infected host, and (L) log transformed viral abundance for the 19° C acclimated/19° C infected host. Dashed lines (A,D,G,J) represent $P_{R,R}$/2. Solid lines (A,D,G,J) represent $P_{R,R}$x2. Closed circles represents experimental data, solid gold line represents model with greatest likelihood, solid blue/red line represents models with lower likelihood (B,C,E,F,H,I,K,L).

**Supplemental Table 5**: AntiSMASH annotated similar gene clusters in *M. aeruginosa* NIES-298 to strain PCC7806. Seven gene clusters found in *M. aeruginosa* NIES-298 along with their location and percent similarity to the most similar known cluster in *M. aeruginosa* PCC7806. Detection was set to “strict” to perform the antiSMASH analysis.

| Type | From - To | Most Similar Known Cluster | Similarity |
| --- | --- | --- | --- |
| Non-ribosomal peptide synthetase, Type I PKS (Polyketide synthase), Non-ribosomal peptide synthetase-like | 1,705,472 - 1,796,003 | microcystin LR | 76% |
| Non-ribosomal peptide synthetase-like, Type I PKS (Polyketide synthase),  Type III PKS (Polyketide synthase) | 2,055,659 - 2,111,667 | merocyclophane C/merocyclophane D | 55% |
| Non-ribosomal peptide synthetase , Non-ribosomal peptide synthetase-like | 2,137,623 - 2,201,347 | aeruginosin 98-A/aeruginosin 98-B/aeruginosin 98-C | 85% |
| cyanobactin | 2,526,123 - 2,560,447 | microcyclamide | 88% |
| Non-ribosomal peptide synthetase-like , Type I PKS (Polyketide synthase) | 3,386,143 -  3.434.944 | 1-heptadecene | 100% |
| microviridin | 4,036,793 - 4,056,945 | microviridin B | 100% |
| Non-ribosomal peptide synthetase, microviridin | 4,142,860 -  4,208,136 | micropeptin K139 | 87% |

**Supplemental Figure 13:** Secondary metabolites differentially expressed dependent on temperature in wild type *M. aeruginosa* PCC7806. **“**Core” genes are shown for secondary metabolite gene clusters that were differentially expressed dependent on culture incubation temperature (26° C or 19° C). “Core” genes are defined as biosynthetic genes that are essential for the production of the secondary metabolite. “Core” genes in the clusters: aeruginosin (complete read), microcyclamide (complete read), and micropeptin (incomplete read: ¾ core genes) are represented. The x-axis is time in hours. The y-axis is transcripts per million (TPM). It should be noted that the y-axis has different scaling for each subplot in the figure. Blue shading represents times at which chemostat cultures were incubated at 19° C. Above each subplot is a representation of each corresponding gene cluster with plotted “core” genes color coordinated to the points on the plot. Genes colored grey correspond to additional genes not essential to production of the secondary metabolite. Each point is the average of two replicates.


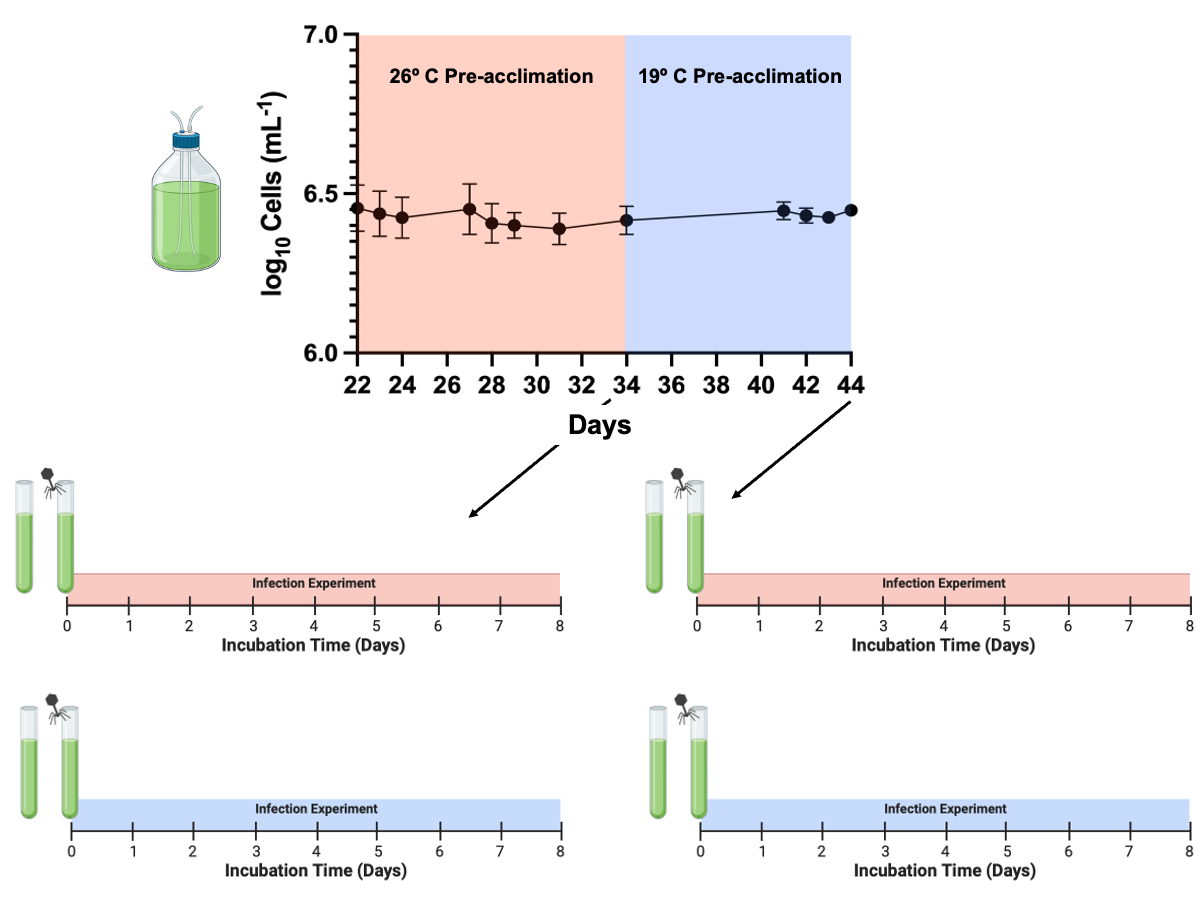


**Supplemental Figure 14:** Schematic of experimental methods. Axenic *M. aeruginosa* NIES-298 was pre-acclimated in chemostat culture at 26° C and 19° C until steady state was reached as determined by flow cytometry algal cell concentrations. Once steady state was reached at each temperature condition (26° C = day 34; 19° C = day 44) samples were taken for infection experiments which were performed at both 26° C and 19° C.
